# Supplementary material for: National Early Warning Scores and COVID-19 deaths in care homes: an ecological time-series study
Source: BMJ Open. 2021 Sep 13;11(9):e045579. doi: 10.1136/bmjopen-2020-045579 (PMC8438578; doi:10.1136/bmjopen-2020-045579)

Supplemental information

Supplemental table 1: Filter values for biologically implausible NEWS component measurements

Measurement	Lower limit	Upper limit	Total (n)	n exceeding lower limit	%	n exceeding upper limit	%
Temperature (C)	>=32	<=41	3087	12	0.4	28	0.9
Pulse rate (beats/minute)	>=17	<=250	3087	10	0.3	148	4.8
Systolic Blood pressure (mmHg)	>=50	<=250	3087	2	0.1	27	0.9
Respiratory rate (breaths/minute)	>=5	<=60	3087	90	2.9	88	2.9
Oxygen saturation (%)	>=45		3087	36	1.2	-	-

Supplemental Figure 1: Cross-correlation function plots for NEWS (and component values) versus all cause deaths in care homes in matched geographical areas

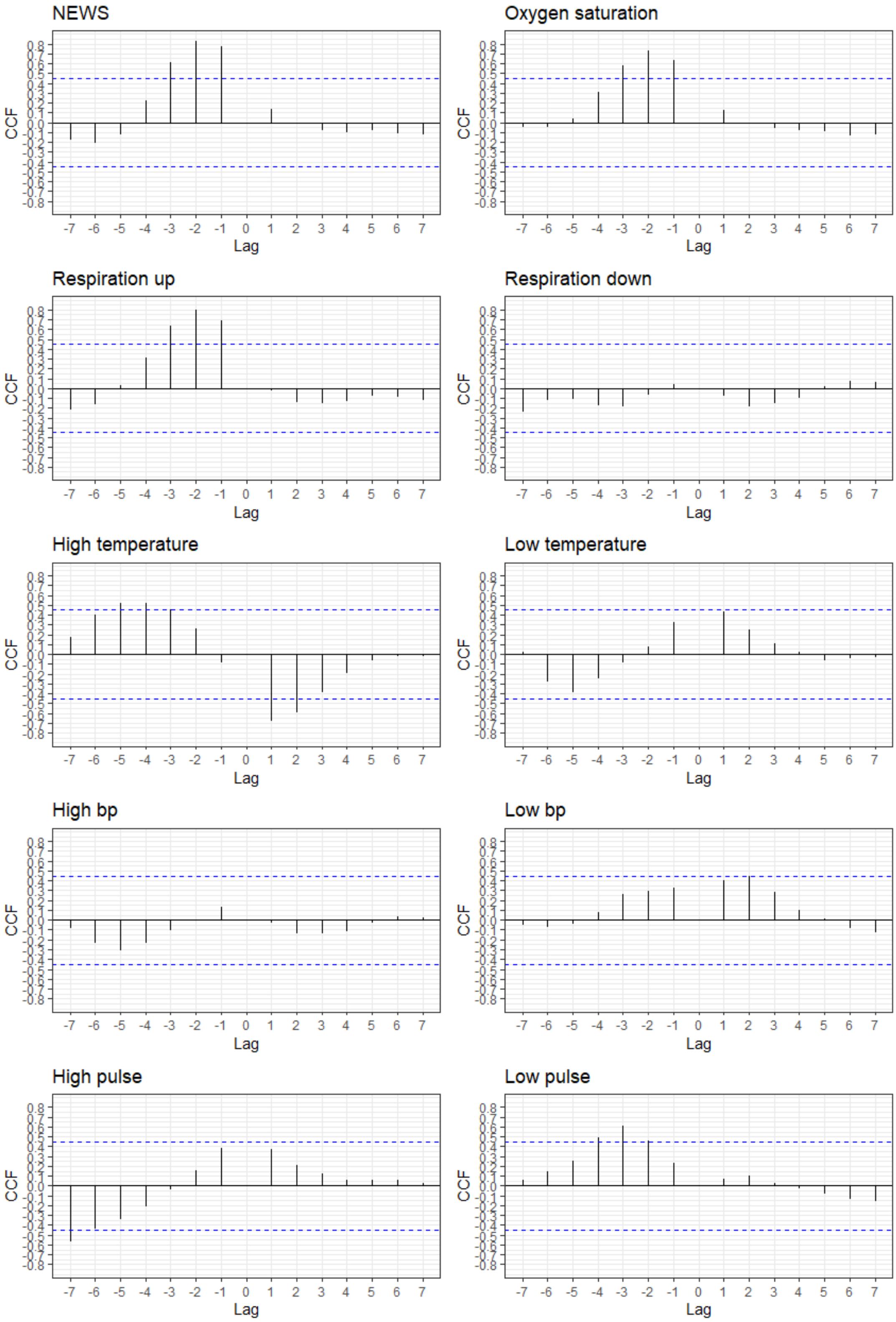

Supplement: Supplementary data [file bmjopen-2020-045579supp001.pdf]
