# Supplementary material for: National Early Warning Scores and COVID-19 deaths in care homes: an ecological time-series study
Source: BMJ Open. 2021 Sep 13;11(9):e045579. doi: 10.1136/bmjopen-2020-045579 (PMC8438578; doi:10.1136/bmjopen-2020-045579)
Supplement: Author's manuscript [file bmjopen-2020-045579.draft_revisions.pdf]

BMJ Open is committed to open peer review. As part of this commitment we make the peer review history of every article we publish publicly available.

When an article is published we post the peer reviewers' comments and the authors' responses online. We also post the versions of the paper that were used during peer review. These are the versions that the peer review comments apply to.

The versions of the paper that follow are the versions that were submitted during the peer review process. They are not the versions of record or the final published versions. They should not be cited or distributed as the published version of this manuscript.

BMJ Open is an open access journal and the full, final, typeset and author-corrected version of record of the manuscript is available on our site with no access controls, subscription charges or pay-per-view fees (http://bmjopen.bmj.com).

If you have any questions on BMJ Open's open peer review process please email info.bmjopen@bmj.com

BMJ Open

National Early Warning Scores and COVID-19 deaths in care homes: an ecological time series study

Journal:	*BMJ Open*
Manuscript ID	bmjopen-2020-045579
Article Type:	Original research
Date Submitted by the Author:	06-Oct-2020
Complete List of Authors:	Stow, Daniel; Newcastle University, Population and Health Sciences Institute<br>Barker, Robert; Newcastle University, Population and Health Sciences Institute<br>Matthews, Fiona; Newcastle University, Population Health Sciences Institute<br>Hanratty, Barbara; Newcastle University, Population and Health Sciences
Keywords:	EPIDEMIOLOGY, INFECTIOUS DISEASES, PUBLIC HEALTH, GERIATRIC MEDICINE

SCHOLARONE™
Manuscripts

I, the Submitting Author has the right to grant and does grant on behalf of all authors of the Work (as defined in the below author licence), an exclusive licence and/or a non-exclusive licence for contributions from authors who are: i) UK Crown employees; ii) where BMJ has agreed a CC-BY licence shall apply, and/or iii) in accordance with the terms applicable for US Federal Government officers or employees acting as part of their official duties; on a worldwide, perpetual, irrevocable, royalty-free basis to BMJ Publishing Group Ltd ("BMJ") its licensees and where the relevant Journal is co-owned by BMJ to the co-owners of the Journal, to publish the Work in this journal and any other BMJ products and to exploit all rights, as set out in our licence.

The Submitting Author accepts and understands that any supply made under these terms is made by BMJ to the Submitting Author unless you are acting as an employee on behalf of your employer or a postgraduate student of an affiliated institution which is paying any applicable article publishing charge ("APC") for Open Access articles. Where the Submitting Author wishes to make the Work available on an Open Access basis (and intends to pay the relevant APC), the terms of reuse of such Open Access shall be governed by a Creative Commons licence – details of these licences and which Creative Commons licence will apply to this Work are set out in our licence referred to above.

Other than as permitted in any relevant BMJ Author's Self Archiving Policies, I confirm this Work has not been accepted for publication elsewhere, is not being considered for publication elsewhere and does not duplicate material already published. I confirm all authors consent to publication of this Work and authorise the granting of this licence.

For peer review only

Title

National Early Warning Scores and COVID-19 deaths in care homes: an ecological time series study

# Corresponding author

Daniel Stow (PhD): Research Associate - Daniel.Stow@ncl.ac.uk

Authors

Daniel Stow, Robert O Barker, Fiona E Matthews, Barbara Hanratty

Daniel Stow (PhD): Research Associate - Daniel.Stow@ncl.ac.uk

Population and Health Sciences Institute, Newcastle University, Newcastle upon Tyne, UK, NE2 4AX – Tel: 0191 208 1202

Robert O Barker (MPH): NIHR In Practice Fellow - Robert.Barker@newcastle.ac.uk

Population and Health Sciences Institute, Newcastle University, Newcastle upon Tyne, UK, NE2 4AX

Fiona E Matthews (PhD): Professor of Epidemiology - Fiona.Matthews@newcastle.ac.uk

Population and Health Sciences Institute, Newcastle University, Newcastle upon Tyne, UK, NE2 4AX

Barbara Hanratty (MD): Professor of Primary Care and Public Health - Barbara.Hanratty@newcastle.ac.uk

Population and Health Sciences Institute, Newcastle University, Newcastle upon Tyne, UK, NE2 4AX

Word count

2150 words (1 table, 2 figures, 1 supplemental table, 1 supplemental figure)

Key words

Pandemic, Surveillance, Care Homes, COVID-19

Contribution statement

Contributors: BH and ROB conceived the study. BH ROB and DS acquired and managed the data. DS analysed the data and created the figures. DS, ROB, FEM and BH interpreted the results. DS, BH and ROB drafted the first version of the manuscript. BH and FEM supervised the work. DS is the guarantor and accepts full responsibility for the work and the conduct of the study, had access to the data, and controlled the decision to publish. All co-authors provided critical comments and approved the final version of the manuscript. The corresponding author attests that all listed authors meet authorship criteria and that no others meeting the criteria have been omitted.

For peer review only

Abstract

Background: Tracking COVID-19 infections in the care home population has been challenging, because of the limited availability of testing and varied disease presentation. We consider whether National Early Warning Scores (NEWS/NEWS2) could contribute to COVID-19 surveillance in care homes.

Methods: We analysed NEWS measurements from care homes in England (December 2019 to May 2020). We estimated pre-COVID (baseline) levels for NEWS and NEWS components using 80th and 20th centile scores for measurements before March 2020. We used time-series to compare the proportion of weekly above-baseline NEWS to area-matched reports of registered deaths in care home residents from the Office for National Statistics

Results: 29,656 anonymised NEWS were available from 6,464 people in 480 care home units across 46 local authority areas. From March 23rd to May 20th, there were 5,753 deaths (1,532 involving COVID-19, 4,221 other causes) in corresponding geographical areas. We observed a rise in the proportion of above-baseline NEWS beginning March 16th 2020. The proportion of above-baseline oxygen saturation, respiratory rate and temperature measurements also increased approximately two weeks before peaks in deaths.

Conclusions: NEWS could contribute to disease surveillance in care homes during the COVID-19 pandemic. Oxygen saturation, respiratory rate and temperature could be prioritised as they appear to signal rise in mortality almost as well as NEWS. This study reinforces the need to collate data from care homes, to monitor and protect residents' health. Further work using individual level outcome data is needed to evaluate the role of NEWS in the early detection of resident illness.

For peer review only

Article Summary

Strengths and limitations of the study

- This is one of the few studies providing population level surveillance information on the care home population during the initial peak of infections from COVID-19.
- This study uses widely available public health mortality information, combined with individual level heath observations (National Early Warning Scores), that could be useful for population-level disease surveillance during future waves of COVID-19 infection in care homes.
- The ecological study design was used as a pragmatic approach to make best use of available data: but this is not a causal study, nor a study of diagnostic accuracy, and it is liable to the ecological fallacy.
- Further research using individual level information on mortality and diagnoses, linked to NEWS, is required to evaluate the role of NEWS in assessing individual residents with suspected COVID-19 infection.

For peer review only

Introduction

Care homes have experienced high rates of COVID-19 infection and death. In England, over half of excess mortality in the first months of 2020 is estimated to have been in this setting.[1] Surveillance of COVID-19 in care homes has been difficult, because of a paucity of testing, and the lack of experience with how this disease presents in older people.[2]

An increasing number of UK care homes are collecting data on the National Early Warning Score (NEWS). This is a 'track and trigger' system used in hospitals in the United Kingdom to identify patients at risk of acute deterioration. NEWS requires the measurement of six parameters: temperature, pulse, systolic blood pressure, respiratory rate and oxygen saturation, along with conscious level. The score generated should trigger a pre-specified response, ranging from repeating the score within a specific timeframe, to seeking urgent medical attention.[3] The British Geriatric Society produced guidelines for managing COVID-19 in care homes for older people. To support triage, they recommend training staff to measure a resident's vital signs (temperature, blood pressure, heart rate, pulse oximetry and respiratory rate) when COVID-19 infection is suspected.[4] The risks and benefits of this approach are unknown. In community settings, elevated NEWS scores have been associated with prompt review by a health professional and poor health outcomes.[5] Evidence to support the use of NEWS in care homes is limited, and whether it can support care home staff to identify or predict deterioration in the health of residents is unclear.[6 7, 8] NEWS observations, particularly measurement of blood pressure, require close contact between residents and care home workers, which may transmit COVID-19 infection.

This study will address the dearth of published evidence on the use of NEWS in any setting during the COVID-19 pandemic.[9] The aim is to describe change in NEWS and its components over time and align these data with COVID-19 and all-cause mortality in care homes in England. We address the question of whether NEWS can contribute to surveillance during the pandemic, and whether an abbreviated NEWS (excluding one or more of the component measures) would suffice.

For peer review only

Methods

Study design and setting

We conducted an ecological study by aggregating individual level data for the exposure of interest (NEWS) and comparing these results to area level aggregate data for the outcome of interest (all cause and COVID-19 mortality). Participants were all residents of care homes utilising the same commercial software with cloud storage of data, and they all had at least one NEWS recording.[10] We did not apply any further population inclusion/exclusion criteria.

Exposure information

Anonymised person-level NEWS (and the slightly modified NEWS2) information were obtained from 01/12/2019-20/05/2020 including the individual component measures of NEWS: blood pressure, respiratory rate, pulse rate, temperature, and oxygen saturation. Demographic information on the care home resident (age and sex) were obtained, along with a geographical identifier to examine regional variation. NEWS recordings were made by care home staff using a specific, commercially available package, designed to reduce measurement error.

Outcome measurement

Geographical death data were obtained from Office for National Statistics (ONS) weekly reported registered deaths in care homes due to COVID-19, and all cause deaths (excluding COVID-19) available from week 1 (beginning 29/12/2019), to week 19 (ending 10/05/2020).[11] ONS reporting areas and care home geographical labels were mapped as closely as possible.

Analysis.

We established baseline levels for NEWS and its component observations, in our population using centile cut points in a random subset of 70% of data collected between 12/2019 and 01/03/2020 (prior to the likely outbreak of COVID-19 in care homes in England). These cut points were then validated in the remaining 30% of observations made before March 2020. NEWS scores above the 80th centile score were defined as above-baseline. For individual

For peer review only

physiological observations, upper and lower thresholds were established using the 80th and 20th centile measurements in the same random subset (we calculated the lowest 20th centile only for oxygen saturation). These cut points represent markers of increase in each parameter at a population level and are not measures of individual clinical concern. We removed biologically implausible values from the dataset before creating the centile cut points (Supplemental Table 1). We used quantile regression to measure the impact of age and sex on centile scores but did not find evidence to support age/sex specific 20th and 80th centile cut points in this population (details available from the authors on request).

We calculated the proportion of above-baseline NEWS measurements and the component observations, on a weekly basis aggregated across all geographical areas providing data. We plotted the proportion of weekly above-baseline measures in participating care homes as a time series against the weekly number of care home deaths due to COVID-19 and all-cause mortality (excluding COVID-19) occurring in the matched geographical areas present in our data. We calculated the cross-correlation between above-baseline NEWS scores and daily deaths (all causes) for time lags between zero and seven weeks. The individual physiological observations that anticipated COVID-19 mortality trends were combined to see if these could be useful for collection instead of the full NEWS panel.

All data management and analyses were conducted using R version 3.6.3[12] we used the ccf function in the stats R package to calculate cross-correlations.

Patient and public involvement

This was a study in response to a Public Health Emergency of International Concern. Patients or the public were not involved in the design, conduct, or reporting of this rapid response research.

For peer review only

Results

Care home population

Care home data were available from 6,464 individuals, 2,007 men (mean age 80.1 years, SD=12.6) and 3,373 women (mean age 83.0 years, SD=12.9). Information on gender was missing from 1,086 (16.8%) people, and age information was missing for 116 (1.8%) people. 441 biologically implausible NEWS component scores were removed from the dataset (supplemental table 1).

Geographical variation in reporting

29,656 NEWS recordings were made across 46 Local Authority (LA) areas, from 480 unique care home IDs (identifiers for the device used to record the measurement, representing a care home, or a distinct unit within a care home). Most recordings were made in two LAs in the north east of England (n=11,029 and n=10,347), and in one London borough (n=3,411).

Deaths in care homes

There were 10,407 registered deaths in care homes in the 46 LA and CCG areas between 29/12/2019 and 10/05/2020. The first death from COVID-19 was registered in week commencing 23/03/2020. From 23/03/2020 to 10/05/2020, there were 5,753 deaths of care home residents - 1,532 with an underlying cause of COVID-19 and 4,221 due to causes excluding COVID-19.

Baseline NEWS centile scores

Table 1 contains information on NEWS taken from 9,586 (70% subset of 13,694) recordings made before 01/03/2020. Table 1 also contains thresholds for NEWS 20th centile scores, and 80th and 20th centile scores for NEWS components calculated in this subset, and the proportion of observations exceeding these values in the 30% validation dataset.

For peer review only

Table 1: NEWS values before March 2020 in the development dataset, cut points for baseline measurements and proportion of above baseline measurements in the validation dataset

	Development dataset						Validation dataset	
Measurement	median	min	max	IQR*	20th centile	80th centile	% below 20th centile	% above 80th centile
NEWS	2.0	0.0	15.0	3.0		>4		14.7%
Temperature (C)	36.5	32.0	40.0	0.6	<36.2	>=36.9	18.4%	25.8%
Pulse rate (beats/min)	76.0	22.0	212.0	19.0	<66	>=89	19.6%	20.0%
Systolic BP (mmHg)	123.0	50.0	235.0	26.0	<109	>=143	20.5%	19.4%
Respiratory rate (breaths/min)	19.0	6.0	60.0	3.0	<17	>=22	19.4%	18.3%
Oxygen saturation (%)	95.0	47.0	100.0	4.0	<=92		17.6%	

*IQR - Interquartile range

NEWS measurements and care home deaths

The proportion of above baseline NEWS observations was stable from week 1 (30/12/2019 – 05/01/ 2020) until week 12 (16/03/2020-22/03/2020) and week 13 (23/03/2020-29/03/2020) when there was a marked increase. This increase happened in the weeks before the majority of COVID-19 and non-COVID-19 deaths began to occur (from week 15 (06/04 – 12/04). The proportion of above baseline NEWS scores peaked in week 15, before beginning to decline again from week 16 (13/04-19/04) onwards (figure 1). The highest correlation was observed for a two-week lag ($r=0.82$, $p \leq 0.05$, supplemental figure 1)

Individual NEWS component measures and care home deaths

The proportion of above baseline measures of high respiratory rate ($r=0.73$, $p \leq 0.05$ for a two-week lag) and low oxygen saturation ($r=0.80$, $p \leq 0.05$ for a two-week lag) appear to follow the pattern of COVID-19 and non-COVID-19 deaths more closely than other component measures (supplemental figure 1). The proportion of above baseline measures of temperature appeared to be decreasing between week 0 and week 10, before rising slightly to plateau until week 15 before declining again.

For peer review only

Figure 1: The proportion of above baseline NEWS and component measurements from December 2019 to May 2020 (lines), compared to care home deaths in corresponding geographical areas in England (bars).

FIGURE 1 HERE

Combination of NEWS component measures

Figure 2 shows paired combinations of above baseline respiratory rate and temperature and below baseline oxygen saturation. All increase just before peaks in COVID-19 and non COVID-19 deaths (Figure 2).

Figure 2: The proportion of above baseline NEWS component combinations December 2019 to May 2020, compared to care home deaths in corresponding geographical areas in England.

FIGURE 2 HERE

For peer review only

Discussion

This study suggests that NEWS could make a useful contribution to disease surveillance in care homes during the COVID-19 pandemic. A rise in the proportion of above-baseline NEWS was observed from the middle of March 2020, when the incidence of COVID-19 was believed to be rising in the UK. The proportion of above-baseline measurements of oxygen saturation, respiratory rate and temperature also increased approximately two weeks before peaks in care home deaths in corresponding geographical areas. Oxygen saturation and respiratory rate appear to signal rise in mortality almost as well as total NEWS and may be safer and more practical to measure during a pandemic.

In this study, we observed a two-week time lag between peaks in NEWS measures and deaths. This is similar to the observed time between symptom onset and COVID-19 death in other settings.[13, 14] Evidence for the role of NEWS in acute illness in community settings is growing,[15 16 17 18 19] but to date, only one descriptive study has provided empirical data on NEWS in care homes.[6] A recent systematic review on the use of NEWS in assessing unwell COVID-19 patients in primary care suggested that enthusiasm for its use may be premature.[9] Limitations of this study mean that we cannot draw direct conclusions about the role of NEWS (or its component parameters) in the care of individual residents: further study would be needed to address this question. However, it suggests a role for NEWS in health surveillance at a care home population level. Measurement and monitoring of some NEWS components may be useful in detecting future waves of COVID-19 infection in care homes. Emerging evidence suggests that up to half of care home residents do not have symptoms at the time they test positive for COVID-19.[20] Whether NEWS measurement would detect approaching illness in any of these residents is unknown, and we do not believe it should substitute for a comprehensive programme of testing in care homes. Recent work has shown the value of routinely collected information to target resources during the COVID-19 pandemic,[21] and our study strengthens the case for further work to evaluate the role of NEWS in the care of individual residents.

For peer review only

Strengths and limitations

To our knowledge, this is the first study to examine variation in NEWS in care homes over time. We have described trends over time using a specific software system to collect data on NEWS in care homes. This means that the distribution of care homes within and between areas is not systematic, as it reflects the market share of the software company and local support for digital data collection in care homes. Most recordings were drawn from the north east of England, and a London borough, but we have no information on the proportion of care home residents in each area that are represented in our dataset. All data were anonymised, and without individual outcome data, we examined patterns in and simple correlations between NEWS and area-level weekly registered death information. We were not able to independently assess or verify the accuracy of NEWS measurements made by care home staff using the specific commercial software, and acknowledge that even where training is sufficient, accuracy of respiration rate judgements in particular can be suboptimal.[22] However, our approach using 20th/80th centile scores will have ameliorated some of the impact of potential inaccuracies, and we caution against using single observations in isolation. Furthermore, we removed a small number of biologically implausible values and provide a summary of the number of values removed in supplemental table 1. This study design was a pragmatic approach that made best use of available data, but it is not a causal study, nor a study of diagnostic accuracy, and it is liable to the ecological fallacy.

Conclusions

NEWS may be useful to monitor health in care homes during the COVID-19 pandemic. Use of a shortened NEWS could be recommended for care home population surveillance where COVID-19 is of primary concern (but not for individual care due to the risk of under-estimating the severity of non-respiratory illness). Oxygen saturation, respiratory rate and temperature provide a similar signal to the complete NEWS. The omission of some components of NEWS, such as blood pressure, minimises contact time between residents and care home staff, potentially reducing infection risk where this is of primary concern. Data on mortality and diagnoses, linked to NEWS, is required to evaluate the role of NEWS in assessing individual residents with suspected COVID-19 infection. Collection and

For peer review only

aggregation of data from care homes would facilitate disease surveillance; we argue that introduction of a care home minimum dataset should be a priority for the UK.

Acknowledgements

We are grateful to Solcom (Wyzan), who provided the anonymised NEWS information used in this study, the care home staff who collected the data, and the North East and North Cumbria Academic Health Science Network who support digital data collection in north east care homes.

Conflicts of interest

All authors have completed the ICMJE uniform disclosure form at www.icmje.org/coi_disclosure.pdf and declare: no support from any organisation for the submitted work; no financial relationships with any organisations that might have an interest in the submitted work in the previous three years; no other relationships or activities that could appear to have influenced the submitted work.

Ethical approval

This study was approved by a University Research Ethics Committee (Ref. 3297/2020) and was conducted under the Secretary of State's directions under the Control of Patient Information Regulations, following advice from the Health Research Authority.

Data sharing

Data sharing: Information on the deaths of care home residents is feely available from the source cited. Other data may be available upon reasonable request from barbara.hanratty@newcastle.ac.uk.

Funding

DS and ROB are funded by the NIHR School for Primary Care Research, (Launching Fellowship to DS SPCR2020DS, In Practice Fellowship to ROB IPF-2018-12-010). BH is supported by the NIHR North East and North Cumbria Applied Research Collaboration. BH and FEM are funded by the NIHR Policy Research Unit: Older People and Frailty. This paper presents independent research funded by the National Institute for Health Research. The views expressed are those of the author(s) and not necessarily those of the NHS, the NIHR or the Department of Health and Social Care.

For peer review only

Role of the funder

The study funder played no part in the study design; in the collection, analysis, and interpretation of data; in the writing of the report; or in the decision to submit the article for publication.

References

1. Comas-Herrera A, Fernandez J-L. England: Estimates of mortality of care home residents linked to the COVID-19 pandemic. International Long-Term Care Policy Network; 2020. [Available from: https://ltccovid.org/2020/05/12/estimates-of-mortality-of-care-home-residents-linked-to-the-covid-19-pandemic-in-england/ Accessed 2020-05-19]
2. Tay HS, Harwood R. Atypical presentation of COVID-19 in a frail older person. Age and Ageing. 2020. DOI 10.1093/ageing/afaa068
3. Royal College of Physicians. National Early Warning Score (NEWS) 2 2020 [Available from: https://www.rcplondon.ac.uk/projects/outputs/national-early-warning-score-news-2 Accessed 2020-05-26]
4. British Geriatrics Society. COVID-19: Managing the COVID-19 pandemic in care homes for older people: good practice guide (last updated 14 April 2020) 2020 [Available from: https://www.bgs.org.uk/resources/covid-19-managing-the-covid-19-pandemic-in-care-homes#_edn5 Accessed 30/04/2020]
5. Scott LJ, Redmond NM, Tavaré A, et al. Association between National Early Warning Scores in primary care and clinical outcomes: an observational study in UK primary and secondary care. British Journal of General Practice. 2020:bjgp20X709337. DOI 10.3399/bjgp20X709337
6. Barker RO, Stocker R, Russell S, et al. Distribution of the National Early Warning Score (NEWS) in care home residents. Age and Ageing. 2019;49(1):141-5. DOI 10.1093/ageing/afz130
7. Hodge S, Thompson C, Gordon AL. National early warning scores in care homes: do policy imperatives reflect a genuine need? Age and Ageing. 2019;49(1):5-6. DOI 10.1093/ageing/afz149
8. Smith GB, Prytherch DR, Schmidt PE, et al. Should age be included as a component of track and trigger systems used to identify sick adult patients? Resuscitation. 2008;78(2):109-15. DOI 10.1016/j.resuscitation.2008.03.004
9. Greenhalgh T, Treadwell J, Burrow R, et al. NEWS (or NEWS2) score when assessing possible COVID-19 patients in primary care? Oxford: The Centre for Evidence-Based Medicine; 2020. [Available from: https://www.cebm.net/covid-19/should-we-use-the-news-or-news2-score-when-assessing-patients-with-possible-covid-19-in-primary-care/ Accessed 2020-05-21]
10. Whzan Digital Health. Early warning health detection systems 2020 [Available from: https://www.whzan.uk/ Accessed 2020-05-20]
11. Office of National Statistics. Deaths involving COVID-19 in the care sector, England and Wales: deaths occurring up to 1 May 2020 and registered up to 9 May 2020 (provisional): ONS; 2020 [Available from: https://www.ons.gov.uk/peoplepopulationandcommunity/birthsdeathsandmarriages/deaths/articles/deathsinvolvingcovid19inthecaresectorenglandandwales/deathsoccurringupto1may2020andregisteredupto9may2020provisional Accessed 2020-05-18]
12. R Core Team. R: A language and environment for statistical computing. Vienna, Austria: R Foundation for Statistical Computing; 2020.
13. Chen T, Wu D, Chen H, et al. Clinical characteristics of 113 deceased patients with coronavirus disease 2019: retrospective study. BMJ. 2020;368:m1091. DOI 10.1136/bmj.m1091

For peer review only

14. Verity R, Okell LC, Dorigatti I, et al. Estimates of the severity of coronavirus disease 2019: a model-based analysis. The Lancet Infectious Diseases. DOI 10.1016/S1473-3099(20)30243-7
15. Finnikin S, Hayward G, Wilson F, Lasserson D. Are referrals to hospital from out-of-hours primary care associated with National Early Warning Scores? Emergency Medicine Journal. 2020;37(5):279. DOI 10.1136/emermed-2019-209069
16. Scott LJ, Redmond NM, Garrett J, et al. Distributions of the National Early Warning Score (NEWS) across a healthcare system following a large-scale roll-out. Emergency Medicine Journal. 2019;36(5):287. DOI 10.1136/emermed-2018-208140
17. Brangan E, Banks J, Brant H, et al. Using the National Early Warning Score (NEWS) outside acute hospital settings: a qualitative study of staff experiences in the West of England. BMJ Open. 2018;8(10):e022528. DOI 10.1136/bmjopen-2018-022528
18. Silcock DJ, Corfield AR, Gowens PA, Rooney KD. Validation of the National Early Warning Score in the prehospital setting. Resuscitation. 2015;89:31-5. DOI 10.1016/j.resuscitation.2014.12.029
19. Inada-Kim M, Knight T, Sullivan M, et al. The prognostic value of national early warning scores (NEWS) during transfer of care from community settings to hospital: a retrospective service evaluation. BJGP Open. 2020:bjgpopen20X101071. DOI 10.3399/bjgpopen20X101071
20. Graham NSN, Junghans C, Downes R, et al. SARS-CoV-2 infection, clinical features and outcome of COVID-19 in United Kingdom nursing homes. medRxiv. 2020:2020.05.19.20105460. DOI 10.1101/2020.05.19.20105460
21. Thornton J. The “virtual wards” supporting patients with covid-19 in the community. BMJ. 2020;369:m2119. DOI 10.1136/bmj.m2119
22. Latten GHP, Spek M, Muris JWM, et al. Accuracy and interobserver-agreement of respiratory rate measurements by healthcare professionals, and its effect on the outcomes of clinical prediction/diagnostic rules. PLOS ONE. 2019;14(10):e0223155. DOI 10.1371/journal.pone.0223155

For peer review only

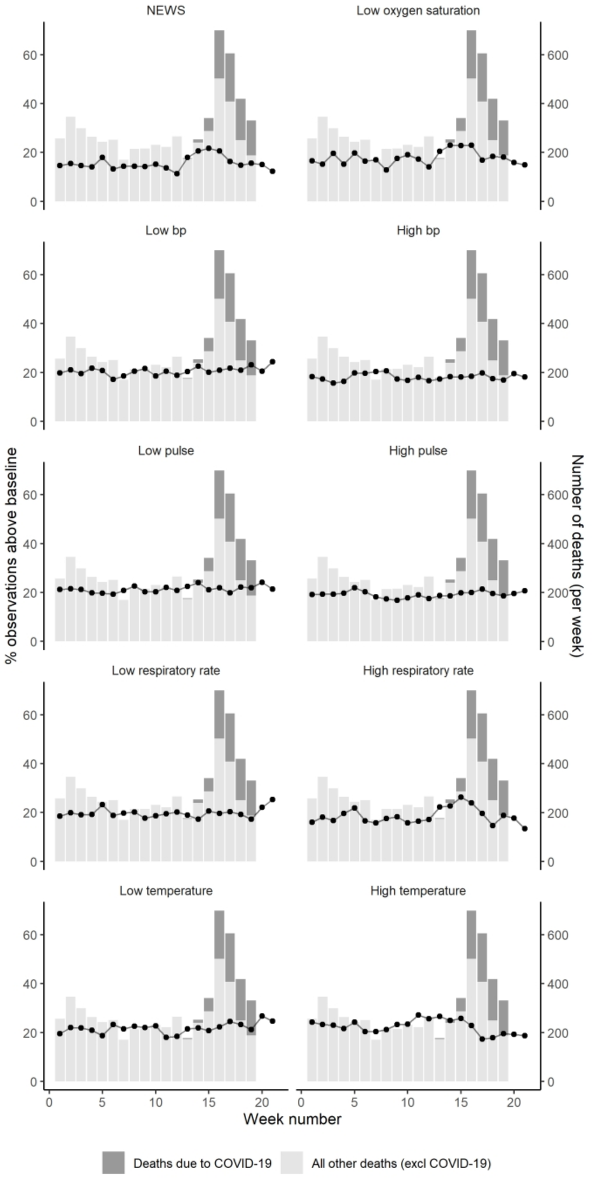

Figure 1: The proportion of above baseline NEWS and component measurements from December 2019 to May 2020 (lines), compared to care home deaths in corresponding geographical areas in England (bars).

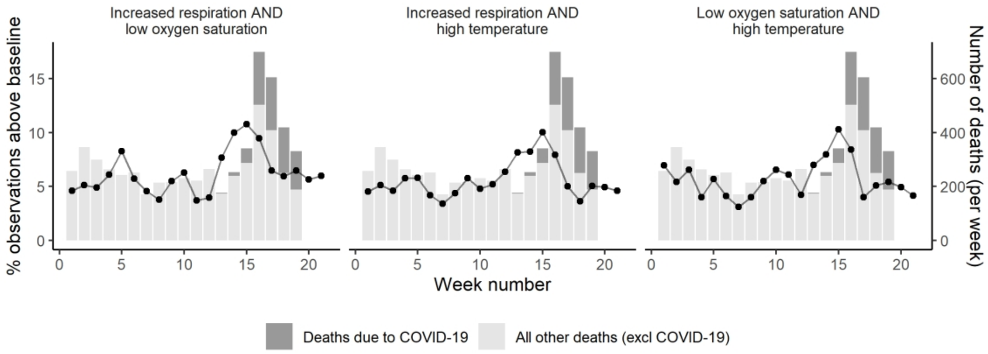

Figure 2: The proportion of above baseline NEWS component combinations December 2019 to May 2020, compared to care home deaths in corresponding geographical areas in England.

Supplemental information

Supplemental table 1: Filter values for biologically implausible NEWS component measurements

Measurement	Lower limit	Upper limit	Total (n)	n exceeding lower limit	%	n exceeding upper limit	%
Temperature (C)	>=32	<=41	3087	12	0.4	28	0.9
Pulse rate (beats/minute)	>=17	<=250	3087	10	0.3	148	4.8
Systolic Blood pressure (mmHg)	>=50	<=250	3087	2	0.1	27	0.9
Respiratory rate (breaths/minute)	>=5	<=60	3087	90	2.9	88	2.9
Oxygen saturation (%)	>=45		3087	36	1.2	-	-

For peer review only

Supplemental Figure 1: Cross-correlation function plots for NEWS (and component values) versus all cause deaths in care homes in matched geographical areas

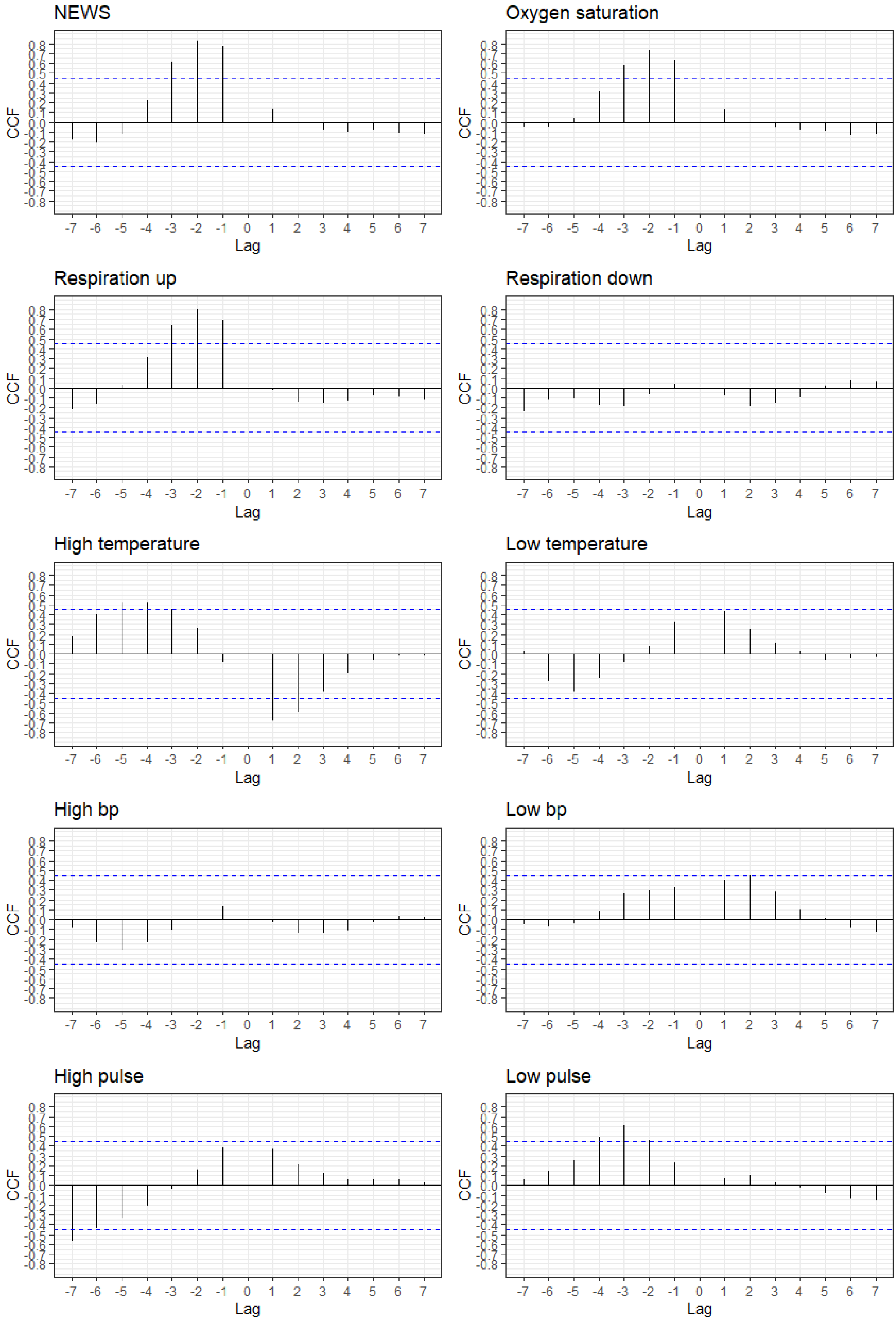

STROBE Statement—checklist of items that should be included in reports of observational studies

	Item No	Recommendation	Page No
**Title and abstract**	1	(*a*) Indicate the study's design with a commonly used term in the title or the abstract	1
		(*b*) Provide in the abstract an informative and balanced summary of what was done and what was found	2
**Introduction**			
Background/rationale	2	Explain the scientific background and rationale for the investigation being reported	4
Objectives	3	State specific objectives, including any prespecified hypotheses	4
**Methods**			
Study design	4	Present key elements of study design early in the paper	5
Setting	5	Describe the setting, locations, and relevant dates, including periods of recruitment, exposure, follow-up, and data collection	5
Participants	6	(*a*) *Cohort study*—Give the eligibility criteria, and the sources and methods of selection of participants. Describe methods of follow-up<br>*Case-control study*—Give the eligibility criteria, and the sources and methods of case ascertainment and control selection. Give the rationale for the choice of cases and controls<br>*Cross-sectional study*—Give the eligibility criteria, and the sources and methods of selection of participants	5
		(*b*) *Cohort study*—For matched studies, give matching criteria and number of exposed and unexposed<br>*Case-control study*—For matched studies, give matching criteria and the number of controls per case	
Variables	7	Clearly define all outcomes, exposures, predictors, potential confounders, and effect modifiers. Give diagnostic criteria, if applicable	5/6
Data sources/ measurement	8*	For each variable of interest, give sources of data and details of methods of assessment (measurement). Describe comparability of assessment methods if there is more than one group	*5/6*
Bias	9	Describe any efforts to address potential sources of bias	5/6
Study size	10	Explain how the study size was arrived at	NA
Quantitative variables	11	Explain how quantitative variables were handled in the analyses. If applicable, describe which groupings were chosen and why	6
Statistical methods	12	(*a*) Describe all statistical methods, including those used to control for confounding	6
		(*b*) Describe any methods used to examine subgroups and interactions	NA
		(*c*) Explain how missing data were addressed	NA
		(*d*) *Cohort study*—If applicable, explain how loss to follow-up was addressed<br>*Case-control study*—If applicable, explain how matching of cases and controls was addressed<br>*Cross-sectional study*—If applicable, describe analytical methods taking account of sampling strategy	NA
		(*e*) Describe any sensitivity analyses	

Continued on next page

For peer review only - http://bmjopen.bmj.com/site/about/guidelines.xhtml

For peer review only

**Results**			
Participants	13*	(a) Report numbers of individuals at each stage of study—eg numbers potentially eligible, examined for eligibility, confirmed eligible, included in the study, completing follow-up, and analysed	7
		(b) Give reasons for non-participation at each stage	NA
		(c) Consider use of a flow diagram	NA
Descriptive data	14*	(a) Give characteristics of study participants (eg demographic, clinical, social) and information on exposures and potential confounders	7
		(b) Indicate number of participants with missing data for each variable of interest	NA
		(c) *Cohort study*—Summarise follow-up time (eg, average and total amount)	NA
Outcome data	15*	*Cohort study*—Report numbers of outcome events or summary measures over time	*7*
		Case-control study—Report numbers in each exposure category, or summary measures of exposure	*NA*
		Cross-sectional study—Report numbers of outcome events or summary measures	*NA*
Main results	16	(*a*) Give unadjusted estimates and, if applicable, confounder-adjusted estimates and their precision (eg, 95% confidence interval). Make clear which confounders were adjusted for and why they were included	8
		(*b*) Report category boundaries when continuous variables were categorized	NA
		(*c*) If relevant, consider translating estimates of relative risk into absolute risk for a meaningful time period	NA
Other analyses	17	Report other analyses done—eg analyses of subgroups and interactions, and sensitivity analyses	9 & 10
**Discussion**			
Key results	18	Summarise key results with reference to study objectives	11
Limitations	19	Discuss limitations of the study, taking into account sources of potential bias or imprecision. Discuss both direction and magnitude of any potential bias	12
Interpretation	20	Give a cautious overall interpretation of results considering objectives, limitations, multiplicity of analyses, results from similar studies, and other relevant evidence	12
Generalisability	21	Discuss the generalisability (external validity) of the study results	12
**Other information**			
Funding	22	Give the source of funding and the role of the funders for the present study and, if applicable, for the original study on which the present article is based	13

*Give information separately for cases and controls in case-control studies and, if applicable, for exposed and unexposed groups in cohort and cross-sectional studies.

Note: An Explanation and Elaboration article discusses each checklist item and gives methodological background and published examples of transparent reporting. The STROBE checklist is best used in conjunction with this article (freely available on the Web sites of PLoS Medicine at http://www.plosmedicine.org/, Annals of Internal Medicine at http://www.annals.org/, and Epidemiology at http://www.epidem.com/). Information on the STROBE Initiative is available at www.strobe-statement.org.

For peer review only

BMJ Open

National Early Warning Scores and COVID-19 deaths in care homes: an ecological time series study

Journal:	*BMJ Open*
Manuscript ID	bmjopen-2020-045579.R1
Article Type:	Original research
Date Submitted by the Author:	01-Feb-2021
Complete List of Authors:	Stow, Daniel; Newcastle University, Population and Health Sciences Institute<br>Barker, Robert; Newcastle University, Population and Health Sciences Institute<br>Matthews, Fiona; Newcastle University, Population Health Sciences Institute<br>Hanratty, Barbara; Newcastle University, Population and Health Sciences
**Primary Subject Heading**:	Public health
Secondary Subject Heading:	Epidemiology, Infectious diseases, Geriatric medicine
Keywords:	EPIDEMIOLOGY, INFECTIOUS DISEASES, PUBLIC HEALTH, GERIATRIC MEDICINE

SCHOLARONE™ Manuscripts

I, the Submitting Author has the right to grant and does grant on behalf of all authors of the Work (as defined in the below author licence), an exclusive licence and/or a non-exclusive licence for contributions from authors who are: i) UK Crown employees; ii) where BMJ has agreed a CC-BY licence shall apply, and/or iii) in accordance with the terms applicable for US Federal Government officers or employees acting as part of their official duties; on a worldwide, perpetual, irrevocable, royalty-free basis to BMJ Publishing Group Ltd ("BMJ") its licensees and where the relevant Journal is co-owned by BMJ to the co-owners of the Journal, to publish the Work in this journal and any other BMJ products and to exploit all rights, as set out in our licence.

The Submitting Author accepts and understands that any supply made under these terms is made by BMJ to the Submitting Author unless you are acting as an employee on behalf of your employer or a postgraduate student of an affiliated institution which is paying any applicable article publishing charge ("APC") for Open Access articles. Where the Submitting Author wishes to make the Work available on an Open Access basis (and intends to pay the relevant APC), the terms of reuse of such Open Access shall be governed by a Creative Commons licence – details of these licences and which Creative Commons licence will apply to this Work are set out in our licence referred to above.

Other than as permitted in any relevant BMJ Author's Self Archiving Policies, I confirm this Work has not been accepted for publication elsewhere, is not being considered for publication elsewhere and does not duplicate material already published. I confirm all authors consent to publication of this Work and authorise the granting of this licence.

For peer review only

Title

National Early Warning Scores and COVID-19 deaths in care homes: an ecological time series study

# Corresponding author

Daniel Stow (PhD): Research Associate - Daniel.Stow@ncl.ac.uk

Authors

Daniel Stow, Robert O Barker, Fiona E Matthews, Barbara Hanratty

Daniel Stow (PhD): Research Associate - Daniel.Stow@ncl.ac.uk

Population and Health Sciences Institute, Newcastle University, Newcastle upon Tyne, UK, NE2 4AX – Tel: 0191 208 1202

Robert O Barker (MPH): NIHR In Practice Fellow - Robert.Barker@newcastle.ac.uk

Population and Health Sciences Institute, Newcastle University, Newcastle upon Tyne, UK, NE2 4AX

Fiona E Matthews (PhD): Professor of Epidemiology - Fiona.Matthews@newcastle.ac.uk

Population and Health Sciences Institute, Newcastle University, Newcastle upon Tyne, UK, NE2 4AX

Barbara Hanratty (MD): Professor of Primary Care and Public Health - Barbara.Hanratty@newcastle.ac.uk

Population and Health Sciences Institute, Newcastle University, Newcastle upon Tyne, UK, NE2 4AX

Word count

2150 words (1 table, 2 figures, 1 supplemental table, 1 supplemental figure)

Key words

Pandemic, Surveillance, Care Homes, COVID-19

For peer review only - http://bmjopen.bmj.com/site/about/guidelines.xhtml

Abstract

Objectives: To investigate whether National Early Warning Scores (NEWS/NEWS2) could contribute to COVID-19 surveillance in care homes.

Setting: 460 Care home units using the same software package to collect data on residents, from 46 local authority areas in England.

Participants: 6,464 care home residents with at least one NEWS recording.

Exposure measure 29,656 anonymised person-level NEWS from 29/12/2019 to 20/05/2020 with component physiological measures: systolic blood pressure, respiratory rate, pulse rate, temperature, and oxygen saturation. Baseline values for each measure calculated using 80th and 20th centile scores before March 2020.

Outcome measure Cross correlation comparison of time series with Office for National Statistics (ONS) weekly reported registered deaths of care home residents where COVID-19 was the underlying cause of death, and all other deaths (excluding COVID-19) up to 10/05/2020.

Results: Deaths due to COVID-19 were registered from 23/03/2020 in the local authority areas represented in the study. Between 23/03/2020 and 10/05/2020, there were 5,753 deaths (1,532 involving COVID-19 and 4,221 other causes). We observed a rise in the proportion of above-baseline NEWS beginning March 16th 2020, followed two weeks later by an increase in registered deaths (cross correlation of r=0.82, p< 0.05 for a two week lag) in corresponding local authorities. The proportion of above-baseline oxygen saturation, respiratory rate and temperature measurements also increased approximately two weeks before peaks in deaths.

Conclusions: NEWS could contribute to COVID-19 disease surveillance in care homes during the pandemic. Oxygen saturation, respiratory rate and temperature could be prioritised as they appear to signal rise in mortality almost as well as NEWS. This study reinforces the need to collate data from care homes, to monitor and protect residents' health. Further work using individual level outcome data is needed to evaluate the role of NEWS in the early detection of resident illness.

For peer review only - http://bmjopen.bmj.com/site/about/guidelines.xhtml

For peer review only

For peer review only - http://bmjopen.bmj.com/site/about/guidelines.xhtml

Article Summary

Strengths and limitations of the study

- This is one of the few studies providing population level surveillance information on the care home population during the initial peak of infections from COVID-19.
- This study uses widely available public health mortality information, combined with individual level health observations based on vital sign measurements (National Early Warning Scores), that could be useful for population-level COVID-19 disease surveillance during future waves of COVID-19 infection in care homes.
- The ecological study design was used as a pragmatic approach to make best use of available data: but this is not a causal study, nor a study of diagnostic accuracy, and it is liable to the ecological fallacy.
- Further research using individual level information on mortality and diagnoses, linked to NEWS, is required to evaluate the role of NEWS in assessing individual residents with suspected COVID-19 infection.

For peer review only

For peer review only - http://bmjopen.bmj.com/site/about/guidelines.xhtml

Introduction

Care homes have experienced high rates of COVID-19 infection and death. In England, over half of excess mortality in the first months of 2020 is estimated to have been in this setting.[1] Surveillance of COVID-19 in care homes has been difficult, because of a paucity of testing, and the lack of experience with how this disease presents in older people.[2]

An increasing number of United Kingdom (UK) care homes now collect National Early Warning Score (NEWS). This tool (originally developed by the Royal College of Physicians in 2012,[3] and updated in 2017)[4] is used in hospitals in the UK to identify patients at risk of acute deterioration and improve patient safety. NEWS requires the measurement of six simple physiological parameters: temperature, pulse, systolic blood pressure, respiratory rate and oxygen saturation, and level of consciousness or new confusion. The score generated should trigger a pre-specified response, ranging from repeating the score within a specific timeframe, to seeking urgent medical attention.[5]

The British Geriatrics Society produced guidelines for managing COVID-19 in care homes for older people. To support triage, they recommend training staff to measure a resident's vital signs (temperature, blood pressure, heart rate, pulse oximetry and respiratory rate) when COVID-19 infection is suspected.[6] The risks and benefits of this approach are unknown. In community settings, elevated NEWS scores have been associated with prompt review by a health professional and poor health outcomes.[7] Evidence to support the use of NEWS in care homes is limited, and whether it can support care home staff to identify or predict deterioration in the health of residents is unclear.[8 9-11] NEWS observations, particularly measurement of blood pressure, require close contact between residents and care home workers, which may transmit COVID-19 infection.

This study will address the dearth of published evidence on the use of NEWS in any setting during the COVID-19 pandemic.[12] The aim is to describe change in NEWS and its components over time and align these data with COVID-19 and all-cause mortality in care homes in England. We address the question of whether NEWS can contribute to surveillance

For peer review only - http://bmjopen.bmj.com/site/about/guidelines.xhtml

during the pandemic, and whether an abbreviated NEWS (excluding one or more of the component measures) would suffice.

For peer review only

For peer review only - http://bmjopen.bmj.com/site/about/guidelines.xhtml

Methods

Study design and setting

We conducted an ecological study by aggregating individual level data for the exposure of interest (NEWS) and comparing these results to area level aggregate data for the outcome of interest (all cause and COVID-19 mortality). Participants were all residents of care homes utilising the same commercial software with cloud storage of data, and they all had at least one NEWS recording.[13] We did not apply any further population inclusion/exclusion criteria.

Exposure information

Anonymised person-level NEWS (and the slightly modified NEWS2)[4] information were obtained from 01/12/2019-20/05/2020 including the individual component vital sign measures of NEWS: blood pressure, respiratory rate, pulse rate, temperature, and oxygen saturation. Demographic information on the care home resident (age and sex) were obtained, along with a geographical identifier to examine regional variation. NEWS recordings were made by care home staff using a specific, commercially available package designed to reduce measurement error. The commercial provider supplies equipment to measure vital signs and record physiological observations, automatically generate NEWS, and upload the data to their cloud storage servers. The system has previously been described elsewhere.[11]

Outcome measurement

Geographical death data were obtained from Office for National Statistics (ONS) weekly reported registered deaths in care homes due to COVID-19, and all cause deaths (excluding COVID-19) available from week 1 (beginning 29/12/2019), to week 19 (ending 10/05/2020).[14] ONS reporting areas and care home geographical labels were mapped as closely as possible.

Analysis.

We established baseline levels for NEWS and its component observations, in our population using centile cut points in a random subset of 70% of data collected between 12/2019 and 01/03/2020 (prior to the likely outbreak of COVID-19 in care homes in England). These cut

For peer review only - http://bmjopen.bmj.com/site/about/guidelines.xhtml

points were then validated in the remaining 30% of observations made before March 2020. NEWS scores above the 80th centile score were defined as above-baseline. For individual physiological observations, upper and lower thresholds were established using the 80th and 20th centile measurements in the same random subset (we calculated the lowest 20th centile only for oxygen saturation: only lower values indicate a health problem). These cut points represent markers of increase in each parameter at a population level and are not measures of clinical concern at the individual resident level, which are defined elsewhere.[4] We removed biologically implausible values from the dataset before creating the centile cut points (Supplemental Table 1). We used quantile regression to measure the impact of age and sex on centile scores but did not find evidence to support age/sex specific 20th and 80th centile cut points in this population (details available from the authors on request).

We calculated the proportion of above-baseline NEWS measurements and the component observations on a weekly basis, aggregated across all geographical areas providing data. We plotted the proportion of weekly above-baseline measures in participating care homes as a time series against the weekly number of care home deaths due to COVID-19 and all-cause mortality (excluding COVID-19) occurring in the matched geographical areas present in our data.

We calculated the cross-correlation between the two time series (above-baseline NEWS and component scores, and daily registered all cause deaths including COVID-19) for time lags between zero and seven weeks. We combined the individual physiological observations that anticipated COVID-19 mortality trends to see if a 'minimum panel' could be useful for collection instead of the full NEWS panel. This makes our findings relevant to care home settings where NEWS is not calculated, or where all component physiological measures are not performed. Measurement of fewer components is expected to reduce contact time between carers and residents and decrease spread of COVID-19 infection.

All data management and analyses were conducted using R version 3.6.3[15] we used the ccf function in the stats R package to calculate cross-correlations.

For peer review only - http://bmjopen.bmj.com/site/about/guidelines.xhtml

Patient and public involvement

This was a study in response to a Public Health Emergency of International Concern. Patients or the public were not involved in the design, conduct, or reporting of this rapid response research.

For peer review only

For peer review only - http://bmjopen.bmj.com/site/about/guidelines.xhtml

Results

Care home population

Care home data were available from 6,464 individuals, 2,007 men (mean age 80.1 years, SD=12.6) and 3,373 women (mean age 83.0 years, SD=12.9). Information on gender was missing from 1,086 (16.8%) people, and age information was missing for 116 (1.8%) people. 441 biologically implausible NEWS component scores were removed from the dataset (supplemental table 1).

Geographical variation in reporting

29,656 NEWS recordings were made across 46 Local Authority (LA) areas, from 480 unique care home IDs (identifiers for the device used to record the measurement, representing a care home, or a distinct unit within a care home). Most recordings were made in two LAs in the north east of England (n=11,029 and n=10,347), and in one London borough (n=3,411).

Deaths in care homes

There were 10,407 registered deaths in care homes in the 46 LA and CCG areas between 29/12/2019 and 10/05/2020. The first death from COVID-19 was registered in week commencing 23/03/2020. From 23/03/2020 to 10/05/2020, there were 5,753 deaths of care home residents - 1,532 with an underlying cause of COVID-19 and 4,221 due to causes excluding COVID-19.

Baseline NEWS centile scores

Table 1 contains information on NEWS taken from 9,586 (70% subset of 13,694) recordings made before 01/03/2020. Table 1 also contains thresholds for NEWS 20th centile scores, and 80th and 20th centile scores for NEWS components calculated in this subset, and the proportion of observations exceeding these values in the 30% validation dataset.

Table 1: NEWS values before March 2020 in the development dataset, cut points for baseline measurements and proportion of above baseline measurements in the validation dataset

	Development dataset	Validation dataset

For peer review only

For peer review only - http://bmjopen.bmj.com/site/about/guidelines.xhtml

Measurement	median	min	max	IQR*	20th centile	80th centile	% below 20th centile	% above 80th centile
NEWS	2.0	0.0	15.0	3.0		>4		14.7%
Temperature (C)	36.5	32.0	40.0	0.6	<36.2	>=36.9	18.4%	25.8%
Pulse rate (beats/min)	76.0	22.0	212.0	19.0	<66	>=89	19.6%	20.0%
Systolic BP (mmHg)	123.0	50.0	235.0	26.0	<109	>=143	20.5%	19.4%
Respiratory rate (breaths/min)	19.0	6.0	60.0	3.0	<17	>=22	19.4%	18.3%
Oxygen saturation (%)	95.0	47.0	100.0	4.0	<=92		17.6%	

*IQR - Interquartile range

NEWS measurements and care home deaths

The proportion of above baseline NEWS observations was stable from week 1 (30/12/2019 – 05/01/ 2020) until week 12 (16/03/2020-22/03/2020) and week 13 (23/03/2020-29/03/2020) when there was a marked increase. This increase happened in the weeks before the majority of COVID-19 and non-COVID-19 deaths began to occur (from week 15 (06/04 – 12/04). The proportion of above baseline NEWS scores peaked in week 15, before beginning to decline again from week 16 (13/04-19/04) onwards (figure 1). The highest correlation was observed for a two-week lag (r=0.82, $p \leq 0.05$, supplemental figure 1)

Individual NEWS component measures and care home deaths

The proportion of above baseline measures of high respiratory rate (r=0.73, $p \leq 0.05$ for a two-week lag) and low oxygen saturation (r=0.80, $p \leq 0.05$ for a two-week lag) appear to follow the pattern of COVID-19 and non-COVID-19 deaths more closely than other component measures (supplemental figure 1). The proportion of above baseline measures of temperature appeared to be decreasing between week 0 and week 10, before rising slightly to plateau until week 15 before declining again.

Figure 1: The proportion of above baseline NEWS and component measurements from December 2019 to May 2020 (lines), compared to care home deaths in corresponding geographical areas in England (bars).

FIGURE 1 HERE

For peer review only - http://bmjopen.bmj.com/site/about/guidelines.xhtml

Combination of NEWS component measures

Figure 2 shows paired combinations of above baseline respiratory rate and temperature and below baseline oxygen saturation. All increase just before peaks in COVID-19 and non COVID-19 deaths (Figure 2).

Figure 2: The proportion of above baseline NEWS component combinations December 2019 to May 2020, compared to care home deaths in corresponding geographical areas in England.

FIGURE 2 HERE

For peer review only

For peer review only - http://bmjopen.bmj.com/site/about/guidelines.xhtml

Discussion

This study suggests that NEWS could make a useful contribution to COVID-19 disease surveillance in care homes during the pandemic. A rise in the proportion of above-baseline NEWS was observed from the middle of March 2020, when the incidence of COVID-19 was believed to be rising in the UK. The proportion of above-baseline measurements of oxygen saturation, respiratory rate and temperature also increased approximately two weeks before peaks in care home deaths in corresponding geographical areas. Oxygen saturation and respiratory rate appear to signal rise in mortality almost as well as total NEWS and may be safer and more practical to measure during a pandemic.

In this study, we observed a two-week time lag between peaks in NEWS measures and deaths. This is similar to the observed time between symptom onset and COVID-19 death in other settings.[16, 17] Evidence for the role of NEWS in acute illness in community settings is growing,[18 19 20 21 11, 22] but to date, only one descriptive study has provided empirical data on NEWS in care homes.[8] A recent systematic review on the use of NEWS in assessing unwell COVID-19 patients in primary care suggested that enthusiasm for its use may be premature.[12] Limitations of this study mean that we cannot draw direct conclusions about the role of NEWS (or its component parameters) in the care of individual residents: further study would be needed to address this question. However, it suggests a role for NEWS in COVID-19 disease surveillance at a care home population level in the pandemic. Measurement and monitoring of some NEWS components may be useful in detecting future waves of COVID-19 infection in care homes. Emerging evidence suggests that up to half of care home residents do not have symptoms at the time they test positive for COVID-19.[23] Whether NEWS measurement could signal impending illness in any of these residents at the individual level is unclear, and we do not believe it should substitute for a comprehensive programme of testing in care homes. Recent work has shown the value of routinely collected information to target resources during the COVID-19 pandemic,[24] and our study strengthens the case for further work to evaluate the role of NEWS in the care of individual residents.

For peer review only - http://bmjopen.bmj.com/site/about/guidelines.xhtml

Strengths and limitations

To our knowledge, this is the first study to examine variation in NEWS in care homes over time. We have described trends over time in NEWS recorded using a specific software system used to collect data on NEWS in some care homes. This means that the distribution of care homes within and between areas is not systematic, as it reflects the market share of the software company and local support for digital data collection in care homes. Most recordings were drawn from the north east of England, and a London borough, but we have no information on the proportion of care home residents in each area that are represented in our dataset. All data were anonymised, and without individual outcome data, we examined patterns in and simple correlations between NEWS and area-level weekly registered death information. We were not able to independently assess or verify the accuracy of NEWS measurements made by care home staff using the specific commercial software, and acknowledge that even where training is sufficient, the accuracy of vital sign measurement such as respiratory rate can be suboptimal.[25] However, our approach using $20^{th}/80^{th}$ centile scores will have ameliorated some of the impact of potential inaccuracies, and we caution against using single observations in isolation. Furthermore, we removed a small number of biologically implausible values and provide a summary of the number of values removed in supplemental table 1. This study design was a pragmatic approach that made best use of available data, but it is not a causal study, nor a study of diagnostic accuracy, and it is liable to the ecological fallacy.

Conclusions

The recording of the NEWS, and the component physiological measures, may make a useful contribution to COVID-19 disease surveillance during the pandemic. Use of a shortened NEWS could be recommended for care home population surveillance where COVID-19 is of primary concern (but not for individual care due to the risk of under-estimating the severity of non-respiratory illness). Oxygen saturation, respiratory rate and temperature provide a similar signal to the complete NEWS. The omission of some components of NEWS, such as blood pressure, minimises contact time between residents and care home staff, potentially reducing infection risk where this is of primary concern. Data on mortality and diagnoses, linked to NEWS, are required to evaluate the role of NEWS in assessing individual residents

For peer review only - http://bmjopen.bmj.com/site/about/guidelines.xhtml

For peer review only

with suspected COVID-19 infection. Collection and aggregation of data from care homes would facilitate disease surveillance; we argue that introduction of a care home minimum dataset should be a priority for the UK.

Contribution statement

Contributors: BH and ROB conceived the study. BH ROB and DS acquired and managed the data. DS analysed the data and created the figures. DS, ROB, FEM and BH interpreted the results. DS, BH and ROB drafted the first version of the manuscript. BH and FEM supervised the work. DS is the guarantor and accepts full responsibility for the work and the conduct of the study, had access to the data, and controlled the decision to publish. All co-authors provided critical comments and approved the final version of the manuscript. The corresponding author attests that all listed authors meet authorship criteria and that no others meeting the criteria have been omitted.

Competing interests

All authors have completed the ICMJE uniform disclosure form at www.icmje.org/coi_disclosure.pdf and declare: no support from any organisation for the submitted work; no financial relationships with any organisations that might have an interest in the submitted work in the previous three years; no other relationships or activities that could appear to have influenced the submitted work.

Funding

DS is funded by the NIHR School for Primary Care Research, (Launching Fellowship to DS SPCR2020DS, ROB by NIHR In Practice Fellowship IPF-2018-12-010). BH is supported by the NIHR North East and North Cumbria Applied Research Collaboration. BH and FEM are funded by the NIHR Policy Research Unit: Older People and Frailty. This paper presents independent research funded by the National Institute for Health Research. The views expressed are those of the author(s) and not necessarily those of the NHS, the NIHR or the Department of Health and Social Care.

Role of the funder

The funders played no part in the study design; in the collection, analysis, and interpretation of data; in the writing of the report; or in the decision to submit the article for publication.

Data sharing statement

For peer review only - http://bmjopen.bmj.com/site/about/guidelines.xhtml

Data sharing: Information on the deaths of care home residents is feely available from the source cited (https://www.ons.gov.uk/peoplepopulationandcommunity/birthsdeathsandmarriages/deaths/articles/deathsinvolvingcovid19inthecaresectorenglandandwales/deathsoccurringupto1may2020andregisteredupto9may2020provisional). Other data may be available upon reasonable request from barbara.hanratty@newcastle.ac.uk.

Acknowledgements

We are grateful to Solcom (Wyzan), who provided the anonymised NEWS information used in this study, the care home staff who collected the data, and the North East and North Cumbria Academic Health Science Network who support digital data collection in north east care homes.

Ethical approval

This study was approved by a University Research Ethics Committee (Ref. 3297/2020) and was conducted under the Secretary of State's directions under the Control of Patient Information Regulations, following advice from the Health Research Authority.

References

1. Comas-Herrera A, Fernandez J-L. England: Estimates of mortality of care home residents linked to the COVID-19 pandemic. International Long-Term Care Policy Network; 2020. [Available from: https://ltccovid.org/2020/05/12/estimates-of-mortality-of-care-home-residents-linked-to-the-covid-19-pandemic-in-england/ Accessed 2020-05-19]
2. Tay HS, Harwood R. Atypical presentation of COVID-19 in a frail older person. Age and Ageing. 2020. DOI 10.1093/ageing/afaa068
3. Physicians RCo. National Early Warning Score (NEWS) Standardising the assessment of acute-illness severity in the NHS. London: Royal College of Physicians; 2012. [Available from: https://www.rcplondon.ac.uk/projects/outputs/national-early-warning-score-news-2 Accessed 19/01/2021]
4. Physicians RCo. National Early Warning Score (NEWS2) Standardising the assessment of acute-illness severity in the NHS. London: Royal College of Physicians; 2017. [Available from: https://www.rcplondon.ac.uk/projects/outputs/national-early-warning-score-news-2 Accessed 19/01/2021]
5. Royal College of Physicians. National Early Warning Score (NEWS) 2 2020 [Available from: https://www.rcplondon.ac.uk/projects/outputs/national-early-warning-score-news-2 Accessed 2020-05-26]
6. British Geriatrics Society. COVID-19: Managing the COVID-19 pandemic in care homes for older people: good practice guide (last updated 14 April 2020) 2020 [Available from:

For peer review only

For peer review only - http://bmjopen.bmj.com/site/about/guidelines.xhtml

https://www.bgs.org.uk/resources/covid-19-managing-the-covid-19-pandemic-in-care-homes#_edn5 Accessed 30/04/2020]

7. Scott LJ, Redmond NM, Tavaré A, et al. Association between National Early Warning Scores in primary care and clinical outcomes: an observational study in UK primary and secondary care. British Journal of General Practice. 2020:bjgp20X709337. DOI 10.3399/bjgp20X709337

8. Barker RO, Stocker R, Russell S, et al. Distribution of the National Early Warning Score (NEWS) in care home residents. Age and Ageing. 2019;49(1):141-5. DOI 10.1093/ageing/afz130

9. Hodge S, Thompson C, Gordon AL. National early warning scores in care homes: do policy imperatives reflect a genuine need? Age and Ageing. 2019;49(1):5-6. DOI 10.1093/ageing/afz149

10. Smith GB, Prytherch DR, Schmidt PE, et al. Should age be included as a component of track and trigger systems used to identify sick adult patients? Resuscitation. 2008;78(2):109-15. DOI 10.1016/j.resuscitation.2008.03.004

11. Russell S, Stocker R, Barker RO, et al. Implementation of the National Early Warning Score in UK care homes: a qualitative evaluation. British Journal of General Practice. 2020;70(700):e793. DOI 10.3399/bjgp20X713069

12. Greenhalgh T, Treadwell J, Burrow R, et al. NEWS (or NEWS2) score when assessing possible COVID-19 patients in primary care? Oxford: The Centre for Evidence-Based Medicine; 2020. [Available from: https://www.cebm.net/covid-19/should-we-use-the-news-or-news2-score-when-assessing-patients-with-possible-covid-19-in-primary-care/ Accessed 2020-05-21]

13. Whzan Digital Health. Early warning health detection systems 2020 [Available from: https://www.whzan.uk/ Accessed 2020-05-20]

14. Office of National Statistics. Deaths involving COVID-19 in the care sector, England and Wales: deaths occurring up to 1 May 2020 and registered up to 9 May 2020 (provisional): ONS; 2020 [Available from: https://www.ons.gov.uk/peoplepopulationandcommunity/birthsdeathsandmarriages/deaths/articles/deathsinvolvingcovid19inthecaresectorenglandandwales/deathsoccurringupto1may2020andregisteredupto9may2020provisional Accessed 2020-05-18]

15. R Core Team. R: A language and environment for statistical computing. Vienna, Austria: R Foundation for Statistical Computing; 2020.

16. Chen T, Wu D, Chen H, et al. Clinical characteristics of 113 deceased patients with coronavirus disease 2019: retrospective study. BMJ. 2020;368:m1091. DOI 10.1136/bmj.m1091

17. Verity R, Okell LC, Dorigatti I, et al. Estimates of the severity of coronavirus disease 2019: a model-based analysis. The Lancet Infectious Diseases. DOI 10.1016/S1473-3099(20)30243-7

18. Finnikin S, Hayward G, Wilson F, Lasserson D. Are referrals to hospital from out-of-hours primary care associated with National Early Warning Scores? Emergency Medicine Journal. 2020;37(5):279. DOI 10.1136/emermed-2019-209069

19. Scott LJ, Redmond NM, Garrett J, et al. Distributions of the National Early Warning Score (NEWS) across a healthcare system following a large-scale roll-out. Emergency Medicine Journal. 2019;36(5):287. DOI 10.1136/emermed-2018-208140

20. Brangan E, Banks J, Brant H, et al. Using the National Early Warning Score (NEWS) outside acute hospital settings: a qualitative study of staff experiences in the West of England. BMJ Open. 2018;8(10):e022528. DOI 10.1136/bmjopen-2018-022528

21. Silcock DJ, Corfield AR, Gowens PA, Rooney KD. Validation of the National Early Warning Score in the prehospital setting. Resuscitation. 2015;89:31-5. DOI 10.1016/j.resuscitation.2014.12.029

22. Inada-Kim M, Knight T, Sullivan M, et al. The prognostic value of national early warning scores (NEWS) during transfer of care from community settings to hospital: a retrospective service evaluation. BJGP Open. 2020:bjgpopen20X101071. DOI 10.3399/bjgpopen20X101071

23. Graham NSN, Junghans C, Downes R, et al. SARS-CoV-2 infection, clinical features and outcome of COVID-19 in United Kingdom nursing homes. medRxiv. 2020:2020.05.19.20105460. DOI 10.1101/2020.05.19.20105460

For peer review only - http://bmjopen.bmj.com/site/about/guidelines.xhtml

24. Thornton J. The “virtual wards” supporting patients with covid-19 in the community. BMJ. 2020;369:m2119. DOI 10.1136/bmj.m2119
25. Latten GHP, Spek M, Muris JWM, et al. Accuracy and interobserver-agreement of respiratory rate measurements by healthcare professionals, and its effect on the outcomes of clinical prediction/diagnostic rules. PLOS ONE. 2019;14(10):e0223155. DOI 10.1371/journal.pone.0223155

For peer review only

For peer review only - http://bmjopen.bmj.com/site/about/guidelines.xhtml

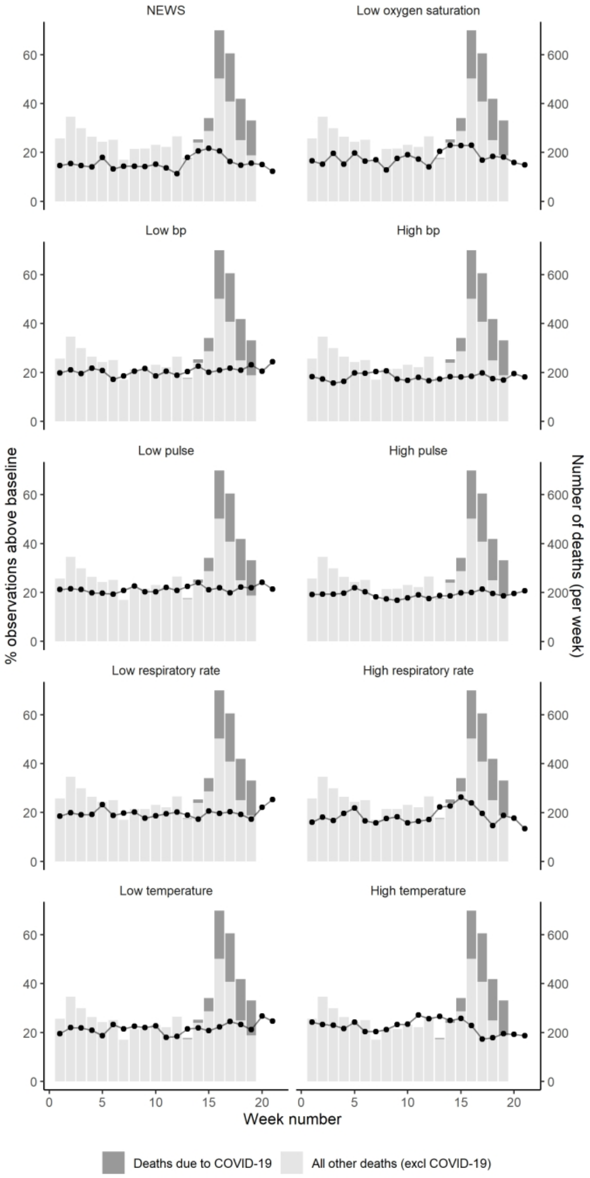

Figure 1: The proportion of above baseline NEWS and component measurements from December 2019 to May 2020 (lines), compared to care home deaths in corresponding geographical areas in England (bars).

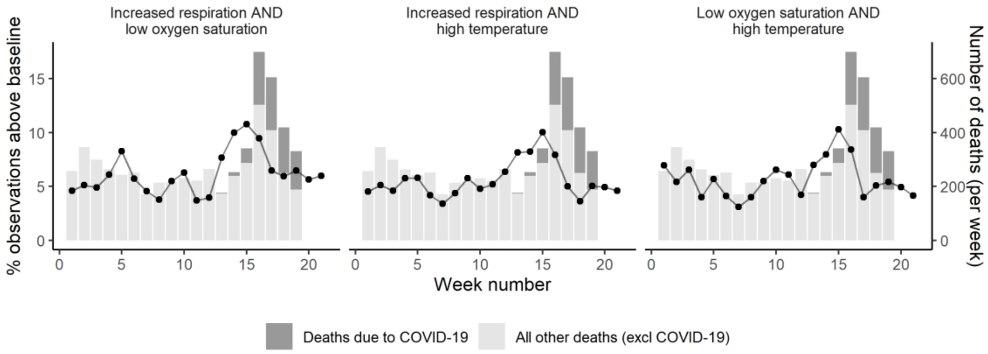

Figure 2: The proportion of above baseline NEWS component combinations December 2019 to May 2020, compared to care home deaths in corresponding geographical areas in England.

Supplemental information

Supplemental table 1: Filter values for biologically implausible NEWS component measurements

Measurement	Lower limit	Upper limit	Total (n)	n exceeding lower limit	%	n exceeding upper limit	%
Temperature (C)	>=32	<=41	3087	12	0.4	28	0.9
Pulse rate (beats/minute)	>=17	<=250	3087	10	0.3	148	4.8
Systolic Blood pressure (mmHg)	>=50	<=250	3087	2	0.1	27	0.9
Respiratory rate (breaths/minute)	>=5	<=60	3087	90	2.9	88	2.9
Oxygen saturation (%)	>=45		3087	36	1.2	-	-

For peer review only

Supplemental Figure 1: Cross-correlation function plots for NEWS (and component values) versus all cause deaths in care homes in matched geographical areas

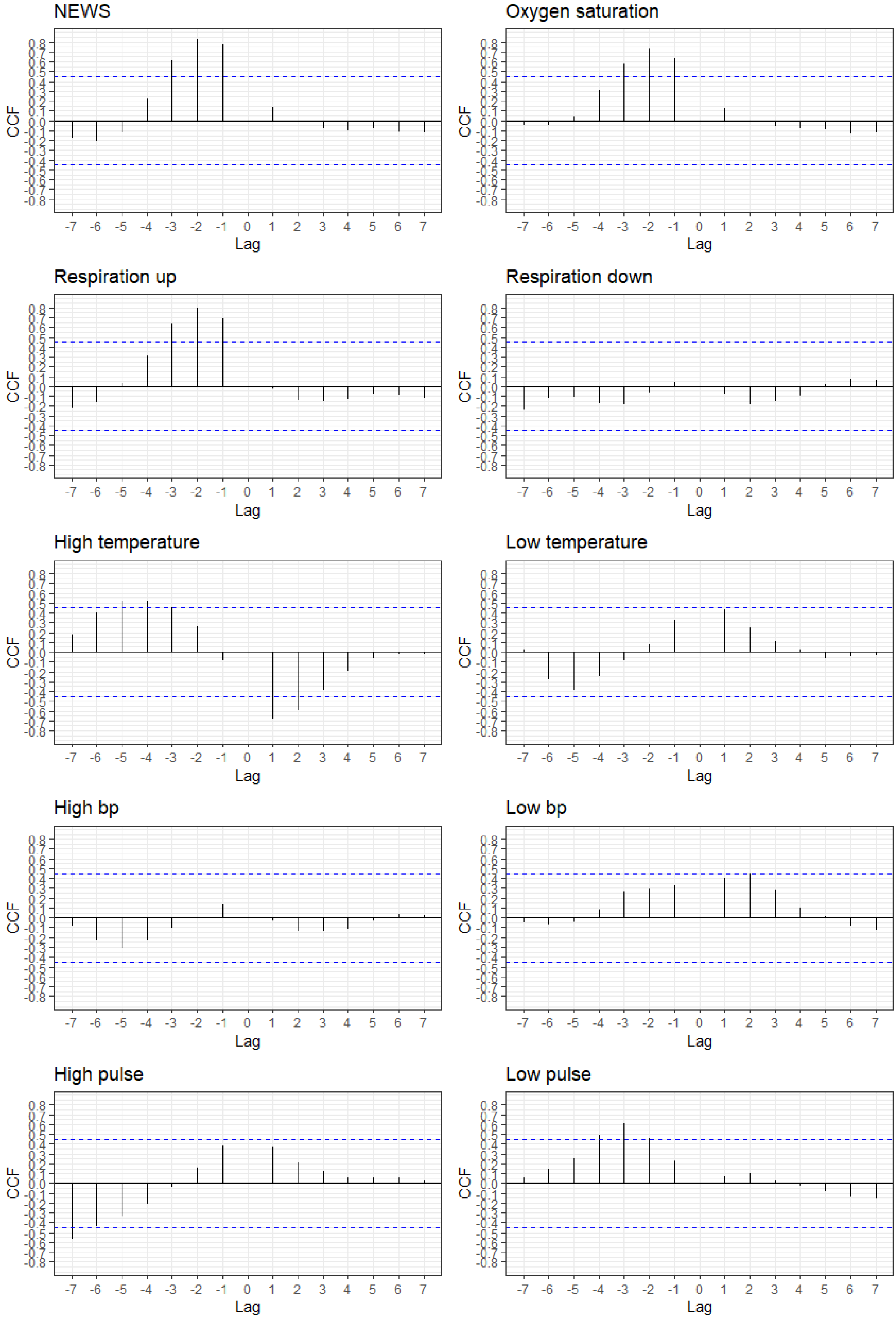

STROBE Statement—checklist of items that should be included in reports of observational studies

	Item No	Recommendation	Page No
**Title and abstract**	1	(*a*) Indicate the study's design with a commonly used term in the title or the abstract	1
		(*b*) Provide in the abstract an informative and balanced summary of what was done and what was found	2
**Introduction**			
Background/rationale	2	Explain the scientific background and rationale for the investigation being reported	4
Objectives	3	State specific objectives, including any prespecified hypotheses	4
**Methods**			
Study design	4	Present key elements of study design early in the paper	5
Setting	5	Describe the setting, locations, and relevant dates, including periods of recruitment, exposure, follow-up, and data collection	5
Participants	6	(*a*) *Cohort study*—Give the eligibility criteria, and the sources and methods of selection of participants. Describe methods of follow-up<br>*Case-control study*—Give the eligibility criteria, and the sources and methods of case ascertainment and control selection. Give the rationale for the choice of cases and controls<br>*Cross-sectional study*—Give the eligibility criteria, and the sources and methods of selection of participants	5
		(*b*) *Cohort study*—For matched studies, give matching criteria and number of exposed and unexposed<br>*Case-control study*—For matched studies, give matching criteria and the number of controls per case	
Variables	7	Clearly define all outcomes, exposures, predictors, potential confounders, and effect modifiers. Give diagnostic criteria, if applicable	5/6
Data sources/ measurement	8*	For each variable of interest, give sources of data and details of methods of assessment (measurement). Describe comparability of assessment methods if there is more than one group	*5/6*
Bias	9	Describe any efforts to address potential sources of bias	5/6
Study size	10	Explain how the study size was arrived at	NA
Quantitative variables	11	Explain how quantitative variables were handled in the analyses. If applicable, describe which groupings were chosen and why	6
Statistical methods	12	(*a*) Describe all statistical methods, including those used to control for confounding	6
		(*b*) Describe any methods used to examine subgroups and interactions	NA
		(*c*) Explain how missing data were addressed	NA
		(*d*) *Cohort study*—If applicable, explain how loss to follow-up was addressed<br>*Case-control study*—If applicable, explain how matching of cases and controls was addressed<br>*Cross-sectional study*—If applicable, describe analytical methods taking account of sampling strategy	NA
		(*e*) Describe any sensitivity analyses	

Continued on next page

For peer review only

Section	Item	Recommendation	Page
**Results**			
Participants	13*	(a) Report numbers of individuals at each stage of study—eg numbers potentially eligible, examined for eligibility, confirmed eligible, included in the study, completing follow-up, and analysed	7
		(b) Give reasons for non-participation at each stage	NA
		(c) Consider use of a flow diagram	NA
Descriptive data	14*	(a) Give characteristics of study participants (eg demographic, clinical, social) and information on exposures and potential confounders	7
		(b) Indicate number of participants with missing data for each variable of interest	NA
		(c) *Cohort study*—Summarise follow-up time (eg, average and total amount)	NA
Outcome data	15*	*Cohort study*—Report numbers of outcome events or summary measures over time	*7*
		Case-control study—Report numbers in each exposure category, or summary measures of exposure	*NA*
		Cross-sectional study—Report numbers of outcome events or summary measures	*NA*
Main results	16	(*a*) Give unadjusted estimates and, if applicable, confounder-adjusted estimates and their precision (eg, 95% confidence interval). Make clear which confounders were adjusted for and why they were included	8
		(*b*) Report category boundaries when continuous variables were categorized	NA
		(*c*) If relevant, consider translating estimates of relative risk into absolute risk for a meaningful time period	NA
Other analyses	17	Report other analyses done—eg analyses of subgroups and interactions, and sensitivity analyses	9 & 10
**Discussion**			
Key results	18	Summarise key results with reference to study objectives	11
Limitations	19	Discuss limitations of the study, taking into account sources of potential bias or imprecision. Discuss both direction and magnitude of any potential bias	12
Interpretation	20	Give a cautious overall interpretation of results considering objectives, limitations, multiplicity of analyses, results from similar studies, and other relevant evidence	12
Generalisability	21	Discuss the generalisability (external validity) of the study results	12
**Other information**			
Funding	22	Give the source of funding and the role of the funders for the present study and, if applicable, for the original study on which the present article is based	13

*Give information separately for cases and controls in case-control studies and, if applicable, for exposed and unexposed groups in cohort and cross-sectional studies.

Note: An Explanation and Elaboration article discusses each checklist item and gives methodological background and published examples of transparent reporting. The STROBE checklist is best used in conjunction with this article (freely available on the Web sites of PLoS Medicine at http://www.plosmedicine.org/, Annals of Internal Medicine at http://www.annals.org/, and Epidemiology at http://www.epidem.com/). Information on the STROBE Initiative is available at www.strobe-statement.org.

For peer review only - http://bmjopen.bmj.com/site/about/guidelines.xhtml

BMJ Open

National Early Warning Scores and COVID-19 deaths in care homes: an ecological time series study

Journal:	*BMJ Open*
Manuscript ID	bmjopen-2020-045579.R2
Article Type:	Original research
Date Submitted by the Author:	15-Jul-2021

Complete List of Authors:	Stow, Daniel; Newcastle University, Population and Health Sciences Institute<br>Barker, Robert; Newcastle University, Population and Health Sciences Institute<br>Matthews, Fiona; Newcastle University, Population Health Sciences Institute<br>Hanratty, Barbara; Newcastle University, Population and Health Sciences

**Primary Subject Heading**:	Public health
Secondary Subject Heading:	Epidemiology, Infectious diseases, Geriatric medicine
Keywords:	EPIDEMIOLOGY, INFECTIOUS DISEASES, PUBLIC HEALTH, GERIATRIC MEDICINE

SCHOLARONE™ Manuscripts

I, the Submitting Author has the right to grant and does grant on behalf of all authors of the Work (as defined in the below author licence), an exclusive licence and/or a non-exclusive licence for contributions from authors who are: i) UK Crown employees; ii) where BMJ has agreed a CC-BY licence shall apply, and/or iii) in accordance with the terms applicable for US Federal Government officers or employees acting as part of their official duties; on a worldwide, perpetual, irrevocable, royalty-free basis to BMJ Publishing Group Ltd ("BMJ") its licensees and where the relevant Journal is co-owned by BMJ to the co-owners of the Journal, to publish the Work in this journal and any other BMJ products and to exploit all rights, as set out in our licence.

The Submitting Author accepts and understands that any supply made under these terms is made by BMJ to the Submitting Author unless you are acting as an employee on behalf of your employer or a postgraduate student of an affiliated institution which is paying any applicable article publishing charge ("APC") for Open Access articles. Where the Submitting Author wishes to make the Work available on an Open Access basis (and intends to pay the relevant APC), the terms of reuse of such Open Access shall be governed by a Creative Commons licence – details of these licences and which Creative Commons licence will apply to this Work are set out in our licence referred to above.

Other than as permitted in any relevant BMJ Author's Self Archiving Policies, I confirm this Work has not been accepted for publication elsewhere, is not being considered for publication elsewhere and does not duplicate material already published. I confirm all authors consent to publication of this Work and authorise the granting of this licence.

For peer review only

Title

National Early Warning Scores and COVID-19 deaths in care homes: an ecological time series study

# Corresponding author

Daniel Stow (PhD): Research Associate - Daniel.Stow@ncl.ac.uk

Authors

Daniel Stow, Robert O Barker, Fiona E Matthews, Barbara Hanratty

Daniel Stow (PhD): Research Associate - Daniel.Stow@ncl.ac.uk

Population and Health Sciences Institute, Newcastle University, Newcastle upon Tyne, UK, NE2 4AX – Tel: 0191 208 1202

Robert O Barker (MPH): NIHR In Practice Fellow - Robert.Barker@newcastle.ac.uk

Population and Health Sciences Institute, Newcastle University, Newcastle upon Tyne, UK, NE2 4AX

Fiona E Matthews (PhD): Professor of Epidemiology - Fiona.Matthews@newcastle.ac.uk

Population and Health Sciences Institute, Newcastle University, Newcastle upon Tyne, UK, NE2 4AX

Barbara Hanratty (MD): Professor of Primary Care and Public Health - Barbara.Hanratty@newcastle.ac.uk

Population and Health Sciences Institute, Newcastle University, Newcastle upon Tyne, UK, NE2 4AX

Word count

2150 words (1 table, 2 figures, 1 supplemental table, 1 supplemental figure)

Key words

Pandemic, Surveillance, Care Homes, COVID-19

For peer review only

Abstract

Objectives: To investigate whether National Early Warning Scores (NEWS/NEWS2) could contribute to COVID-19 surveillance in care homes.

Setting: 460 Care home units using the same software package to collect data on residents, from 46 local authority areas in England.

Participants: 6,464 care home residents with at least one NEWS recording.

Exposure measure 29,656 anonymised person-level NEWS from 29/12/2019 to 20/05/2020 with component physiological measures: systolic blood pressure, respiratory rate, pulse rate, temperature, and oxygen saturation. Baseline values for each measure calculated using 80th and 20th centile scores before March 2020.

Outcome measure Cross correlation comparison of time series with Office for National Statistics (ONS) weekly reported registered deaths of care home residents where COVID-19 was the underlying cause of death, and all other deaths (excluding COVID-19) up to 10/05/2020.

Results: Deaths due to COVID-19 were registered from 23/03/2020 in the local authority areas represented in the study. Between 23/03/2020 and 10/05/2020, there were 5,753 deaths (1,532 involving COVID-19 and 4,221 other causes). We observed a rise in the proportion of above-baseline NEWS beginning March 16th 2020, followed two weeks later by an increase in registered deaths (cross correlation of r=0.82, p< 0.05 for a two week lag) in corresponding local authorities. The proportion of above-baseline oxygen saturation, respiratory rate and temperature measurements also increased approximately two weeks before peaks in deaths.

Conclusions: NEWS could contribute to COVID-19 disease surveillance in care homes during the pandemic. Oxygen saturation, respiratory rate and temperature could be prioritised as they appear to signal rise in mortality almost as well as NEWS. This study reinforces the need to collate data from care homes, to monitor and protect residents' health. Further work using individual level outcome data is needed to evaluate the role of NEWS in the early detection of resident illness.

For peer review only - http://bmjopen.bmj.com/site/about/guidelines.xhtml

1

For peer review only

For peer review only - http://bmjopen.bmj.com/site/about/guidelines.xhtml

Article Summary

Strengths and limitations of the study

- This is one of the few studies providing population level surveillance information on the care home population during the initial peak of infections from COVID-19.
- This study uses widely available public health mortality information, combined with individual level health observations based on vital sign measurements (National Early Warning Scores), that could be useful for population-level COVID-19 disease surveillance during future waves of COVID-19 infection in care homes.
- The ecological study design was used as a pragmatic approach to make best use of available data: but this is not a causal study, nor a study of diagnostic accuracy, and it is liable to the ecological fallacy.
- Further research using individual level information on mortality and diagnoses, linked to NEWS, is required to evaluate the role of NEWS in assessing individual residents with suspected COVID-19 infection.

For peer review only - http://bmjopen.bmj.com/site/about/guidelines.xhtml

Introduction

Care homes have experienced high rates of COVID-19 infection and death. In England, over half of excess mortality in the first months of 2020 is estimated to have been in this setting.[1] Surveillance of COVID-19 in care homes has been difficult, because of a paucity of testing, and the lack of experience with how this disease presents in older people.[2]

An increasing number of United Kingdom (UK) care homes now collect National Early Warning Score (NEWS). This tool (originally developed by the Royal College of Physicians in 2012,[3] and updated in 2017)[4] is used in hospitals in the UK to identify patients at risk of acute deterioration and improve patient safety. NEWS requires the measurement of six simple physiological parameters: temperature, pulse, systolic blood pressure, respiratory rate and oxygen saturation, and level of consciousness or new confusion. The score generated should trigger a pre-specified response, ranging from repeating the score within a specific timeframe, to seeking urgent medical attention.[5]

The British Geriatrics Society produced guidelines for managing COVID-19 in care homes for older people. To support triage, they recommend training staff to measure a resident's vital signs (temperature, blood pressure, heart rate, pulse oximetry and respiratory rate) when COVID-19 infection is suspected.[6] The risks and benefits of this approach are unknown. In community settings, elevated NEWS scores have been associated with prompt review by a health professional and poor health outcomes.[7] Evidence to support the use of NEWS in care homes is limited, and whether it can support care home staff to identify or predict deterioration in the health of residents is unclear.[8 9-11] NEWS observations, particularly measurement of blood pressure, require close contact between residents and care home workers, which may transmit COVID-19 infection.

This study will address the dearth of published evidence on the use of NEWS in any setting during the COVID-19 pandemic.[12] The aim is to describe change in NEWS and its components over time and align these data with COVID-19 and all-cause mortality in care homes in England. We address the question of whether NEWS can contribute to surveillance

For peer review only - http://bmjopen.bmj.com/site/about/guidelines.xhtml

during the pandemic, and whether an abbreviated NEWS (excluding one or more of the component measures) would suffice.

For peer review only

For peer review only - http://bmjopen.bmj.com/site/about/guidelines.xhtml

Methods

Study design and setting

We conducted an ecological study by aggregating individual level data for the exposure of interest (NEWS) and comparing these results to area level aggregate data for the outcome of interest (all cause and COVID-19 mortality). Participants were all residents of care homes utilising the same commercial software with cloud storage of data, and they all had at least one NEWS recording.[13] We did not apply any further population inclusion/exclusion criteria.

Exposure information

Anonymised person-level NEWS (and the slightly modified NEWS2)[4] information were obtained from 01/12/2019-20/05/2020 including the individual component vital sign measures of NEWS: blood pressure, respiratory rate, pulse rate, temperature, and oxygen saturation. Demographic information on the care home resident (age and sex) were obtained, along with a geographical identifier to examine regional variation. NEWS recordings were made by care home staff using a specific, commercially available package designed to reduce measurement error. The commercial provider supplies equipment to measure vital signs and record physiological observations, automatically generate NEWS, and upload the data to their cloud storage servers. The system has previously been described elsewhere.[11]

Outcome measurement

Geographical death data were obtained from Office for National Statistics (ONS) weekly reported registered deaths in care homes due to COVID-19, and all cause deaths (excluding COVID-19) available from week 1 (beginning 29/12/2019), to week 19 (ending 10/05/2020).[14] ONS reporting areas and care home geographical labels were mapped as closely as possible.

Analysis.

We established baseline levels for NEWS and its component observations, in our population using centile cut points in a random subset of 70% of data collected between 12/2019 and 01/03/2020 (prior to the likely outbreak of COVID-19 in care homes in England). These cut

For peer review only - http://bmjopen.bmj.com/site/about/guidelines.xhtml

points were then validated in the remaining 30% of observations made before March 2020. NEWS scores above the 80th centile score were defined as above-baseline. For individual physiological observations, upper and lower thresholds were established using the 80th and 20th centile measurements in the same random subset (we calculated the lowest 20th centile only for oxygen saturation: only lower values indicate a health problem). These cut points represent markers of increase in each parameter at a population level and are not measures of clinical concern at the individual resident level, which are defined elsewhere.[4] We removed biologically implausible values from the dataset before creating the centile cut points (Supplemental Table 1). We used quantile regression to measure the impact of age and sex on centile scores but did not find evidence to support age/sex specific 20th and 80th centile cut points in this population (details available from the authors on request).

We calculated the proportion of above-baseline NEWS measurements and the component observations on a weekly basis, aggregated across all geographical areas providing data. We plotted the proportion of weekly above-baseline measures in participating care homes as a time series against the weekly number of care home deaths due to COVID-19 and all-cause mortality (excluding COVID-19) occurring in the matched geographical areas present in our data.

We calculated the cross-correlation between the two time series (above-baseline NEWS and component scores, and daily registered all cause deaths including COVID-19) for time lags between zero and seven weeks. We combined the individual physiological observations that anticipated COVID-19 mortality trends to see if a 'minimum panel' could be useful for collection instead of the full NEWS panel. This makes our findings relevant to care home settings where NEWS is not calculated, or where all component physiological measures are not performed. Measurement of fewer components is expected to reduce contact time between carers and residents and decrease spread of COVID-19 infection.

All data management and analyses were conducted using R version 3.6.3[15] we used the ccf function in the stats R package to calculate cross-correlations.

For peer review only - http://bmjopen.bmj.com/site/about/guidelines.xhtml

Patient and public involvement

This was a study in response to a Public Health Emergency of International Concern. Patients or the public were not involved in the design, conduct, or reporting of this rapid response research.

For peer review only

For peer review only - http://bmjopen.bmj.com/site/about/guidelines.xhtml

Results

Care home population

Care home data were available from 6,464 individuals, 2,007 men (mean age 80.1 years, SD=12.6) and 3,373 women (mean age 83.0 years, SD=12.9). Information on gender was missing from 1,086 (16.8%) people, and age information was missing for 116 (1.8%) people. 441 biologically implausible NEWS component scores were removed from the dataset (supplemental table 1).

Geographical variation in reporting

29,656 NEWS recordings were made across 46 Local Authority (LA) areas, from 480 unique care home IDs (identifiers for the device used to record the measurement, representing a care home, or a distinct unit within a care home). Most recordings were made in two LAs in the north east of England (n=11,029 and n=10,347), and in one London borough (n=3,411).

Deaths in care homes

There were 10,407 registered deaths in care homes in the 46 LA and CCG areas between 29/12/2019 and 10/05/2020. The first death from COVID-19 was registered in week commencing 23/03/2020. From 23/03/2020 to 10/05/2020, there were 5,753 deaths of care home residents - 1,532 with an underlying cause of COVID-19 and 4,221 due to causes excluding COVID-19.

Baseline NEWS centile scores

Table 1 contains information on NEWS taken from 9,586 (70% subset of 13,694) recordings made before 01/03/2020. Table 1 also contains thresholds for NEWS 20th centile scores, and 80th and 20th centile scores for NEWS components calculated in this subset, and the proportion of observations exceeding these values in the 30% validation dataset.

Table 1: NEWS values before March 2020 in the development dataset, cut points for baseline measurements and proportion of above baseline measurements in the validation dataset

Development dataset Validation dataset

For peer review only - http://bmjopen.bmj.com/site/about/guidelines.xhtml

Measurement	median	min	max	IQR*	20th centile	80th centile	% below 20th centile	% above 80th centile
NEWS	2.0	0.0	15.0	3.0		>4		14.7%
Temperature (C)	36.5	32.0	40.0	0.6	<36.2	>=36.9	18.4%	25.8%
Pulse rate (beats/min)	76.0	22.0	212.0	19.0	<66	>=89	19.6%	20.0%
Systolic BP (mmHg)	123.0	50.0	235.0	26.0	<109	>=143	20.5%	19.4%
Respiratory rate (breaths/min)	19.0	6.0	60.0	3.0	<17	>=22	19.4%	18.3%
Oxygen saturation (%)	95.0	47.0	100.0	4.0	<=92		17.6%	

*IQR - Interquartile range

NEWS measurements and care home deaths

The proportion of above baseline NEWS observations was stable from week 1 (30/12/2019 – 05/01/ 2020) until week 12 (16/03/2020-22/03/2020) and week 13 (23/03/2020-29/03/2020) when there was a marked increase. This increase happened in the weeks before the majority of COVID-19 and non-COVID-19 deaths began to occur (from week 15 (06/04 – 12/04). The proportion of above baseline NEWS scores peaked in week 15, before beginning to decline again from week 16 (13/04-19/04) onwards (figure 1). The highest correlation was observed for a two-week lag (r=0.82, $p \leq 0.05$, supplemental figure 1)

Individual NEWS component measures and care home deaths

The proportion of above baseline measures of high respiratory rate (r=0.73, $p \leq 0.05$ for a two-week lag) and low oxygen saturation (r=0.80, $p \leq 0.05$ for a two-week lag) appear to follow the pattern of COVID-19 and non-COVID-19 deaths more closely than other component measures (supplemental figure 1). The proportion of above baseline measures of temperature appeared to be decreasing between week 0 and week 10, before rising slightly to plateau until week 15 before declining again.

Figure 1: The proportion of above baseline NEWS and component measurements from December 2019 to May 2020 (lines), compared to care home deaths in corresponding geographical areas in England (bars).

FIGURE 1 HERE

For peer review only - http://bmjopen.bmj.com/site/about/guidelines.xhtml

Combination of NEWS component measures

Figure 2 shows paired combinations of above baseline respiratory rate and temperature and below baseline oxygen saturation. All increase just before peaks in COVID-19 and non COVID-19 deaths (Figure 2).

Figure 2: The proportion of above baseline NEWS component combinations December 2019 to May 2020, compared to care home deaths in corresponding geographical areas in England.

FIGURE 2 HERE

For peer review only

For peer review only - http://bmjopen.bmj.com/site/about/guidelines.xhtml

Discussion

This study suggests that NEWS could make a useful contribution to COVID-19 disease surveillance in care homes during the pandemic. A rise in the proportion of above-baseline NEWS was observed from the middle of March 2020, when the incidence of COVID-19 was believed to be rising in the UK. The proportion of above-baseline measurements of oxygen saturation, respiratory rate and temperature also increased approximately two weeks before peaks in care home deaths in corresponding geographical areas. Oxygen saturation and respiratory rate appear to signal rise in mortality almost as well as total NEWS and may be safer and more practical to measure during a pandemic.

In this study, we observed a two-week time lag between peaks in NEWS measures and deaths. This is similar to the observed time between symptom onset and COVID-19 death in other settings.[16, 17] Evidence for the role of NEWS in acute illness in community settings is growing,[18 19 20 21 11, 22] but to date, only one descriptive study has provided empirical data on NEWS in care homes.[8] A recent systematic review on the use of NEWS in assessing unwell COVID-19 patients in primary care suggested that enthusiasm for its use may be premature.[12] Limitations of this study mean that we cannot draw direct conclusions about the role of NEWS (or its component parameters) in the care of individual residents: further study would be needed to address this question. However, it suggests a role for NEWS in COVID-19 disease surveillance at a care home population level in the pandemic. Measurement and monitoring of some NEWS components may be useful in detecting future waves of COVID-19 infection in care homes. Emerging evidence suggests that up to half of care home residents do not have symptoms at the time they test positive for COVID-19.[23] Whether NEWS measurement could signal impending illness in any of these residents at the individual level is unclear, and we do not believe it should substitute for a comprehensive programme of testing in care homes. Recent work has shown the value of routinely collected information to target resources during the COVID-19 pandemic,[24] and our study strengthens the case for further work to evaluate the role of NEWS in the care of individual residents.

For peer review only - http://bmjopen.bmj.com/site/about/guidelines.xhtml

Strengths and limitations

To our knowledge, this is the first study to examine variation in NEWS in care homes over time. We have described trends over time in NEWS recorded using a specific software system used to collect data on NEWS in some care homes. This means that the distribution of care homes within and between areas is not systematic, as it reflects the market share of the software company and local support for digital data collection in care homes. Most recordings were drawn from the north east of England, and a London borough, but we have no information on the proportion of care home residents in each area that are represented in our dataset. All data were anonymised, and without individual outcome data, we examined patterns in and simple correlations between NEWS and area-level weekly registered death information. We were not able to independently assess or verify the accuracy of NEWS measurements made by care home staff using the specific commercial software, and acknowledge that even where training is sufficient, the accuracy of vital sign measurement such as respiratory rate can be suboptimal.[25] However, our approach using 20th/80th centile scores will have ameliorated some of the impact of potential inaccuracies, and we caution against using single observations in isolation. Furthermore, we removed a small number of biologically implausible values and provide a summary of the number of values removed in supplemental table 1. This study design was a pragmatic approach that made best use of available data, but it is not a causal study, nor a study of diagnostic accuracy, and it is liable to the ecological fallacy.

Conclusions

The recording of the NEWS, and the component physiological measures, may make a useful contribution to COVID-19 disease surveillance during the pandemic. Use of a shortened NEWS could be recommended for care home population surveillance where COVID-19 is of primary concern (but not for individual care due to the risk of under-estimating the severity of non-respiratory illness). Oxygen saturation, respiratory rate and temperature provide a similar signal to the complete NEWS. The omission of some components of NEWS, such as blood pressure, minimises contact time between residents and care home staff, potentially reducing infection risk where this is of primary concern. Data on mortality and diagnoses, linked to NEWS, are required to evaluate the role of NEWS in assessing individual residents

For peer review only

For peer review only - http://bmjopen.bmj.com/site/about/guidelines.xhtml

with suspected COVID-19 infection. Collection and aggregation of data from care homes would facilitate disease surveillance; we argue that introduction of a care home minimum dataset should be a priority for the UK.

Contribution statement

Contributors: BH and ROB conceived the study. BH ROB and DS acquired and managed the data. DS analysed the data and created the figures. DS, ROB, FEM and BH interpreted the results. DS, BH and ROB drafted the first version of the manuscript. BH and FEM supervised the work. DS is the guarantor and accepts full responsibility for the work and the conduct of the study, had access to the data, and controlled the decision to publish. All co-authors provided critical comments and approved the final version of the manuscript. The corresponding author attests that all listed authors meet authorship criteria and that no others meeting the criteria have been omitted.

Competing interests

All authors have completed the ICMJE uniform disclosure form at www.icmje.org/coi_disclosure.pdf and declare: no support from any organisation for the submitted work; no financial relationships with any organisations that might have an interest in the submitted work in the previous three years; no other relationships or activities that could appear to have influenced the submitted work.

Funding

DS is funded by the NIHR School for Primary Care Research, (Launching Fellowship SPCR-PDF-2020 -161), ROB by an NIHR In Practice Fellowship (IPF-2018-12-010). BH is supported by the NIHR North East and North Cumbria Applied Research Collaboration. BH and FEM are funded by the NIHR Policy Research Unit: Older People and Frailty (PR-PRU-1217-21502). This paper presents independent research funded by the National Institute for Health Research. The views expressed are those of the author(s) and not necessarily those of the NHS, the NIHR or the Department of Health and Social Care.

Role of the funder

The funders played no part in the study design; in the collection, analysis, and interpretation of data; in the writing of the report; or in the decision to submit the article for publication.

Data sharing statement

For peer review only

For peer review only - http://bmjopen.bmj.com/site/about/guidelines.xhtml

Data sharing: Information on the deaths of care home residents is feely available from the source cited (https://www.ons.gov.uk/peoplepopulationandcommunity/birthsdeathsandmarriages/deaths/articles/deathsinvolvingcovid19inthecaresectorenglandandwales/deathsoccurringupto1may2020andregisteredupto9may2020provisional). Other data may be available upon reasonable request from barbara.hanratty@newcastle.ac.uk.

Acknowledgements

We are grateful to Solcom (Wyzan), who provided the anonymised NEWS information used in this study, the care home staff who collected the data, and the North East and North Cumbria Academic Health Science Network who support digital data collection in north east care homes.

Ethical approval

This study was approved by Newcastle University Research Ethics Committee (Ref. 3297/2020) and was conducted under the Secretary of State's directions under the Control of Patient Information Regulations, following advice from the Health Research Authority.

References

1. Comas-Herrera A, Fernandez J-L. England: Estimates of mortality of care home residents linked to the COVID-19 pandemic. International Long-Term Care Policy Network; 2020. [Available from: https://ltccovid.org/2020/05/12/estimates-of-mortality-of-care-home-residents-linked-to-the-covid-19-pandemic-in-england/ Accessed 2020-05-19]
2. Tay HS, Harwood R. Atypical presentation of COVID-19 in a frail older person. Age and Ageing. 2020. DOI 10.1093/ageing/afaa068
3. Physicians RCo. National Early Warning Score (NEWS) Standardising the assessment of acute-illness severity in the NHS. London: Royal College of Physicians; 2012. [Available from: https://www.rcplondon.ac.uk/projects/outputs/national-early-warning-score-news-2 Accessed 19/01/2021]
4. Physicians RCo. National Early Warning Score (NEWS2) Standardising the assessment of acute-illness severity in the NHS. London: Royal College of Physicians; 2017. [Available from: https://www.rcplondon.ac.uk/projects/outputs/national-early-warning-score-news-2 Accessed 19/01/2021]
5. Royal College of Physicians. National Early Warning Score (NEWS) 2 2020 [Available from: https://www.rcplondon.ac.uk/projects/outputs/national-early-warning-score-news-2 Accessed 2020-05-26]
6. British Geriatrics Society. COVID-19: Managing the COVID-19 pandemic in care homes for older people: good practice guide (last updated 14 April 2020) 2020 [Available from:

For peer review only - http://bmjopen.bmj.com/site/about/guidelines.xhtml

https://www.bgs.org.uk/resources/covid-19-managing-the-covid-19-pandemic-in-care-homes#_edn5 Accessed 30/04/2020]

7. Scott LJ, Redmond NM, Tavaré A, et al. Association between National Early Warning Scores in primary care and clinical outcomes: an observational study in UK primary and secondary care. British Journal of General Practice. 2020:bjgp20X709337. DOI 10.3399/bjgp20X709337

8. Barker RO, Stocker R, Russell S, et al. Distribution of the National Early Warning Score (NEWS) in care home residents. Age and Ageing. 2019;49(1):141-5. DOI 10.1093/ageing/afz130

9. Hodge S, Thompson C, Gordon AL. National early warning scores in care homes: do policy imperatives reflect a genuine need? Age and Ageing. 2019;49(1):5-6. DOI 10.1093/ageing/afz149

10. Smith GB, Prytherch DR, Schmidt PE, et al. Should age be included as a component of track and trigger systems used to identify sick adult patients? Resuscitation. 2008;78(2):109-15. DOI 10.1016/j.resuscitation.2008.03.004

11. Russell S, Stocker R, Barker RO, et al. Implementation of the National Early Warning Score in UK care homes: a qualitative evaluation. British Journal of General Practice. 2020;70(700):e793. DOI 10.3399/bjgp20X713069

12. Greenhalgh T, Treadwell J, Burrow R, et al. NEWS (or NEWS2) score when assessing possible COVID-19 patients in primary care? Oxford: The Centre for Evidence-Based Medicine; 2020. [Available from: https://www.cebm.net/covid-19/should-we-use-the-news-or-news2-score-when-assessing-patients-with-possible-covid-19-in-primary-care/ Accessed 2020-05-21]

13. Whzan Digital Health. Early warning health detection systems 2020 [Available from: https://www.whzan.uk/ Accessed 2020-05-20]

14. Office of National Statistics. Deaths involving COVID-19 in the care sector, England and Wales: deaths occurring up to 1 May 2020 and registered up to 9 May 2020 (provisional): ONS; 2020 [Available from: https://www.ons.gov.uk/peoplepopulationandcommunity/birthsdeathsandmarriages/deaths/articles/deathsinvolvingcovid19inthecaresectorenglandandwales/deathsoccurringupto1may2020andregisteredupto9may2020provisional Accessed 2020-05-18]

15. R Core Team. R: A language and environment for statistical computing. Vienna, Austria: R Foundation for Statistical Computing; 2020.

16. Chen T, Wu D, Chen H, et al. Clinical characteristics of 113 deceased patients with coronavirus disease 2019: retrospective study. BMJ. 2020;368:m1091. DOI 10.1136/bmj.m1091

17. Verity R, Okell LC, Dorigatti I, et al. Estimates of the severity of coronavirus disease 2019: a model-based analysis. The Lancet Infectious Diseases. DOI 10.1016/S1473-3099(20)30243-7

18. Finnikin S, Hayward G, Wilson F, Lasserson D. Are referrals to hospital from out-of-hours primary care associated with National Early Warning Scores? Emergency Medicine Journal. 2020;37(5):279. DOI 10.1136/emermed-2019-209069

19. Scott LJ, Redmond NM, Garrett J, et al. Distributions of the National Early Warning Score (NEWS) across a healthcare system following a large-scale roll-out. Emergency Medicine Journal. 2019;36(5):287. DOI 10.1136/emermed-2018-208140

20. Brangan E, Banks J, Brant H, et al. Using the National Early Warning Score (NEWS) outside acute hospital settings: a qualitative study of staff experiences in the West of England. BMJ Open. 2018;8(10):e022528. DOI 10.1136/bmjopen-2018-022528

21. Silcock DJ, Corfield AR, Gowens PA, Rooney KD. Validation of the National Early Warning Score in the prehospital setting. Resuscitation. 2015;89:31-5. DOI 10.1016/j.resuscitation.2014.12.029

22. Inada-Kim M, Knight T, Sullivan M, et al. The prognostic value of national early warning scores (NEWS) during transfer of care from community settings to hospital: a retrospective service evaluation. BJGP Open. 2020:bjgpopen20X101071. DOI 10.3399/bjgpopen20X101071

23. Graham NSN, Junghans C, Downes R, et al. SARS-CoV-2 infection, clinical features and outcome of COVID-19 in United Kingdom nursing homes. medRxiv. 2020:2020.05.19.20105460. DOI 10.1101/2020.05.19.20105460

For peer review only - http://bmjopen.bmj.com/site/about/guidelines.xhtml

24. Thornton J. The “virtual wards” supporting patients with covid-19 in the community. BMJ. 2020;369:m2119. DOI 10.1136/bmj.m2119

25. Latten GHP, Spek M, Muris JWM, et al. Accuracy and interobserver-agreement of respiratory rate measurements by healthcare professionals, and its effect on the outcomes of clinical prediction/diagnostic rules. PLOS ONE. 2019;14(10):e0223155. DOI 10.1371/journal.pone.0223155

For peer review only

For peer review only - http://bmjopen.bmj.com/site/about/guidelines.xhtml

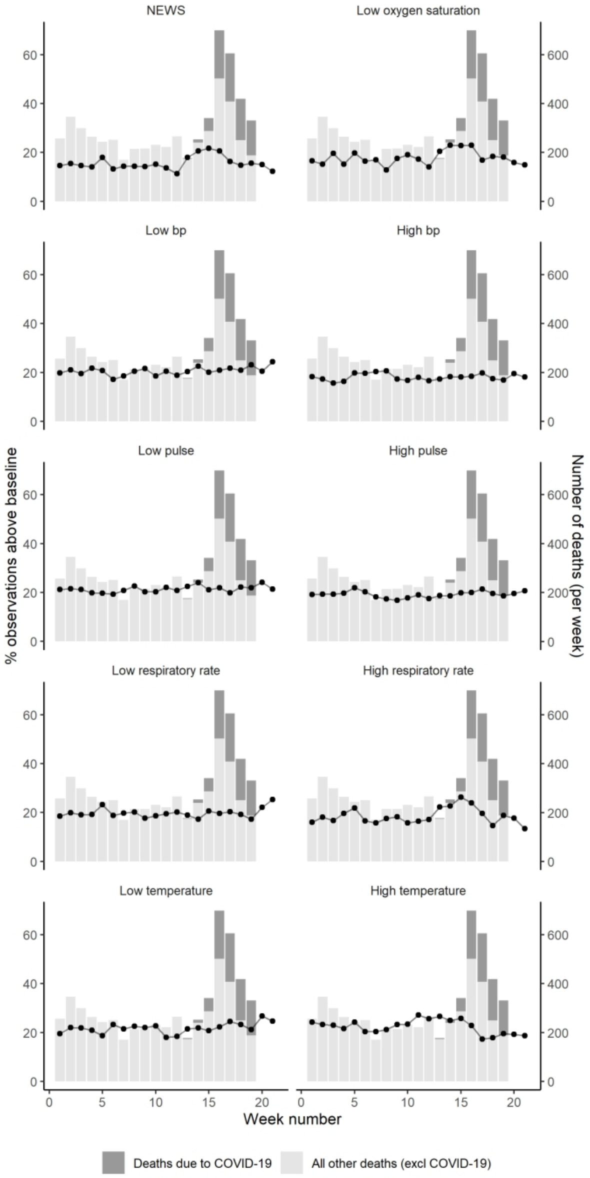

Figure 1: The proportion of above baseline NEWS and component measurements from December 2019 to May 2020 (lines), compared to care home deaths in corresponding geographical areas in England (bars).

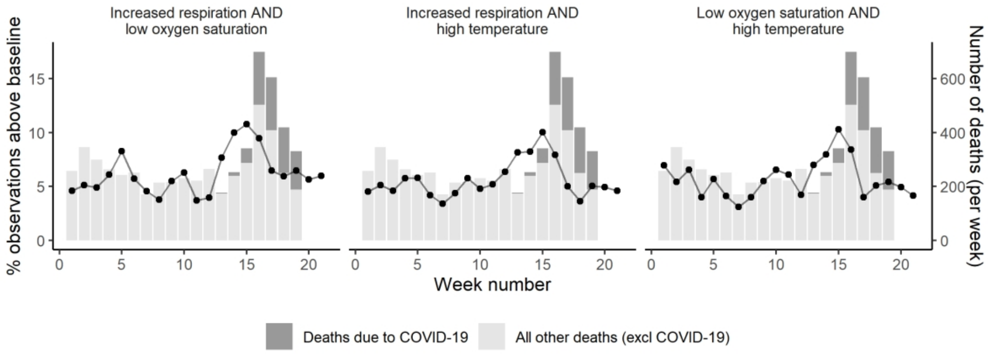

Figure 2: The proportion of above baseline NEWS component combinations December 2019 to May 2020, compared to care home deaths in corresponding geographical areas in England.

Supplemental information

Supplemental table 1: Filter values for biologically implausible NEWS component measurements

Measurement	Lower limit	Upper limit	Total (n)	n exceeding lower limit	%	n exceeding upper limit	%
Temperature (C)	>=32	<=41	3087	12	0.4	28	0.9
Pulse rate (beats/minute)	>=17	<=250	3087	10	0.3	148	4.8
Systolic Blood pressure (mmHg)	>=50	<=250	3087	2	0.1	27	0.9
Respiratory rate (breaths/minute)	>=5	<=60	3087	90	2.9	88	2.9
Oxygen saturation (%)	>=45		3087	36	1.2	-	-

For peer review only

Supplemental Figure 1: Cross-correlation function plots for NEWS (and component values) versus all cause deaths in care homes in matched geographical areas

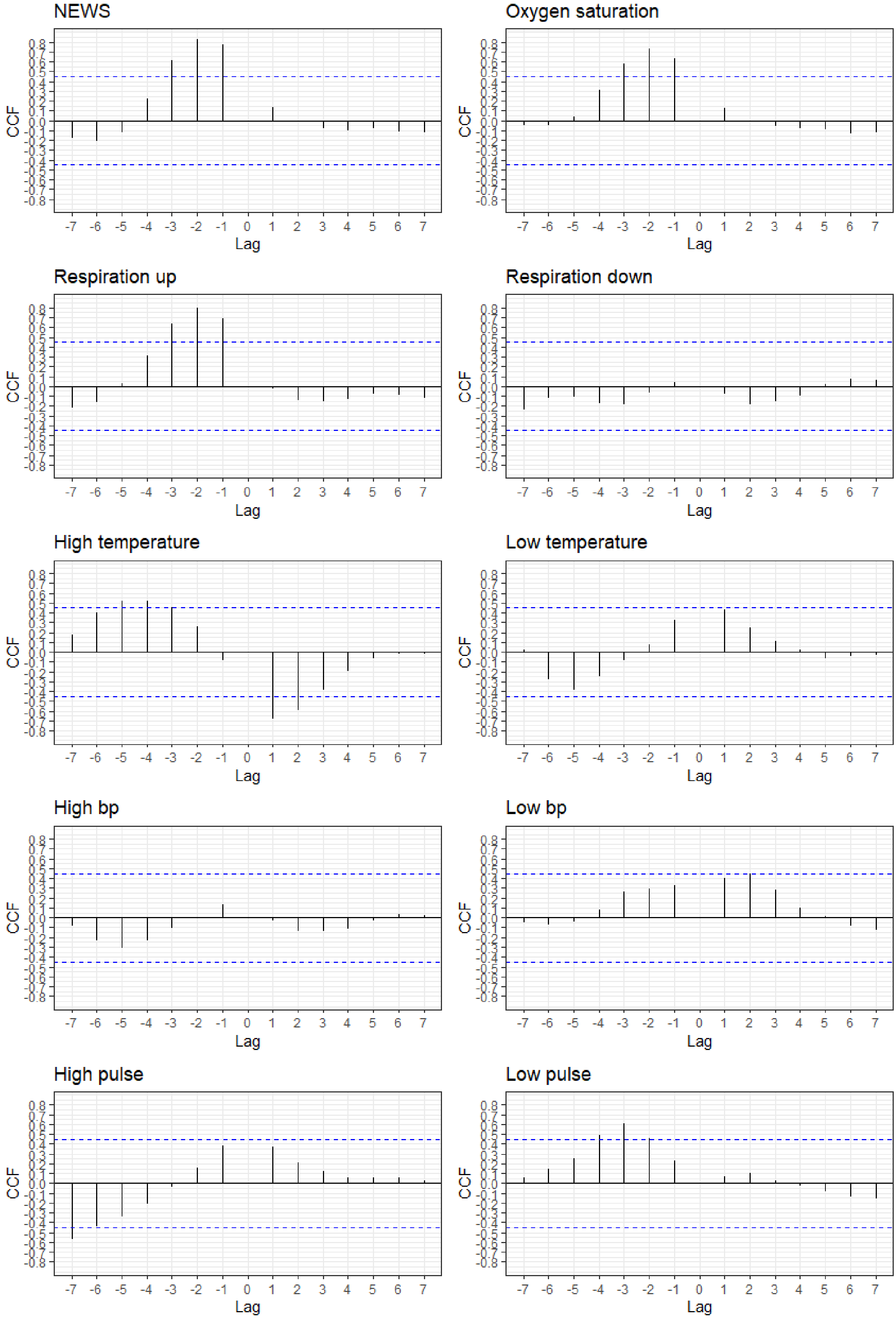

STROBE Statement—checklist of items that should be included in reports of observational studies

	Item No	Recommendation	Page No
**Title and abstract**	1	(*a*) Indicate the study's design with a commonly used term in the title or the abstract	1
		(*b*) Provide in the abstract an informative and balanced summary of what was done and what was found	2
**Introduction**			
Background/rationale	2	Explain the scientific background and rationale for the investigation being reported	4
Objectives	3	State specific objectives, including any prespecified hypotheses	4
**Methods**			
Study design	4	Present key elements of study design early in the paper	5
Setting	5	Describe the setting, locations, and relevant dates, including periods of recruitment, exposure, follow-up, and data collection	5
Participants	6	(*a*) *Cohort study*—Give the eligibility criteria, and the sources and methods of selection of participants. Describe methods of follow-up<br>*Case-control study*—Give the eligibility criteria, and the sources and methods of case ascertainment and control selection. Give the rationale for the choice of cases and controls<br>*Cross-sectional study*—Give the eligibility criteria, and the sources and methods of selection of participants	5
		(*b*) *Cohort study*—For matched studies, give matching criteria and number of exposed and unexposed<br>*Case-control study*—For matched studies, give matching criteria and the number of controls per case	
Variables	7	Clearly define all outcomes, exposures, predictors, potential confounders, and effect modifiers. Give diagnostic criteria, if applicable	5/6
Data sources/ measurement	8*	For each variable of interest, give sources of data and details of methods of assessment (measurement). Describe comparability of assessment methods if there is more than one group	*5/6*
Bias	9	Describe any efforts to address potential sources of bias	5/6
Study size	10	Explain how the study size was arrived at	NA
Quantitative variables	11	Explain how quantitative variables were handled in the analyses. If applicable, describe which groupings were chosen and why	6
Statistical methods	12	(*a*) Describe all statistical methods, including those used to control for confounding	6
		(*b*) Describe any methods used to examine subgroups and interactions	NA
		(*c*) Explain how missing data were addressed	NA
		(*d*) *Cohort study*—If applicable, explain how loss to follow-up was addressed<br>*Case-control study*—If applicable, explain how matching of cases and controls was addressed<br>*Cross-sectional study*—If applicable, describe analytical methods taking account of sampling strategy	NA
		(*e*) Describe any sensitivity analyses	

Continued on next page

For peer review only - http://bmjopen.bmj.com/site/about/guidelines.xhtml

**Results**			
Participants	13*	(a) Report numbers of individuals at each stage of study—eg numbers potentially eligible, examined for eligibility, confirmed eligible, included in the study, completing follow-up, and analysed	7
		(b) Give reasons for non-participation at each stage	NA
		(c) Consider use of a flow diagram	NA
Descriptive data	14*	(a) Give characteristics of study participants (eg demographic, clinical, social) and information on exposures and potential confounders	7
		(b) Indicate number of participants with missing data for each variable of interest	NA
		(c) *Cohort study*—Summarise follow-up time (eg, average and total amount)	NA
Outcome data	15*	*Cohort study*—Report numbers of outcome events or summary measures over time	*7*
		Case-control study—Report numbers in each exposure category, or summary measures of exposure	*NA*
		Cross-sectional study—Report numbers of outcome events or summary measures	*NA*
Main results	16	(*a*) Give unadjusted estimates and, if applicable, confounder-adjusted estimates and their precision (eg, 95% confidence interval). Make clear which confounders were adjusted for and why they were included	8
		(*b*) Report category boundaries when continuous variables were categorized	NA
		(*c*) If relevant, consider translating estimates of relative risk into absolute risk for a meaningful time period	NA
Other analyses	17	Report other analyses done—eg analyses of subgroups and interactions, and sensitivity analyses	9 & 10
**Discussion**			
Key results	18	Summarise key results with reference to study objectives	11
Limitations	19	Discuss limitations of the study, taking into account sources of potential bias or imprecision. Discuss both direction and magnitude of any potential bias	12
Interpretation	20	Give a cautious overall interpretation of results considering objectives, limitations, multiplicity of analyses, results from similar studies, and other relevant evidence	12
Generalisability	21	Discuss the generalisability (external validity) of the study results	12
**Other information**			
Funding	22	Give the source of funding and the role of the funders for the present study and, if applicable, for the original study on which the present article is based	13

*Give information separately for cases and controls in case-control studies and, if applicable, for exposed and unexposed groups in cohort and cross-sectional studies.

Note: An Explanation and Elaboration article discusses each checklist item and gives methodological background and published examples of transparent reporting. The STROBE checklist is best used in conjunction with this article (freely available on the Web sites of PLoS Medicine at http://www.plosmedicine.org/, Annals of Internal Medicine at http://www.annals.org/, and Epidemiology at http://www.epidem.com/). Information on the STROBE Initiative is available at www.strobe-statement.org.

For peer review only
